# Tuning the separation and coupling of corannulene trianion-radicals through sizable alkali metal belts†
†Electronic supplementary information (ESI) available: Details of preparation, characterization, X-ray diffraction study, and theoretical calculations. CCDC 1516728. For ESI and crystallographic data in CIF or other electronic format see DOI: 10.1039/c6sc05370j



**DOI:** 10.1039/c6sc05370j

**Published:** 2017-02-23

**Authors:** Sarah N. Spisak, Andrey Yu. Rogachev, Alexander V. Zabula, Alexander S. Filatov, Rodolphe Clérac, Marina A. Petrukhina

**Affiliations:** a Department of Chemistry , University at Albany , State University of New York , Albany , NY 12222 , USA . Email: mpetrukhina@albany.edu; b Department of Chemistry , Illinois Institute of Technology , Chicago , IL 60616 , USA . Email: arogache@iit.edu; c Department of Chemistry , University of Pennsylvania , Philadelphia , PA 19104 , USA; d CNRS , CRPP , UPR 8641 , F-33600 , Pessac , France; e Univ. Bordeaux , CRPP , UPR 8641 , F-33600 , Pessac , France

## Abstract

The first heterobimetallic sandwich-type aggregate formed by bowl-shaped corannulene trianion-radicals, C_20_H_10_˙^3–^, has been synthesized using mixed-metal reduction of C_20_H_10_. The product was crystallographically characterized to reveal the self-assembly of [Cs^+^//(C_20_H_10_^3–^)/4K^+^/(C_20_H_10_^3–^)//Cs^+^], in which two triply-charged corannulene decks encapsulate a rectangle of four potassium ions (the K···K separations are 4.212(4) and 5.185(4) Å), with the exterior concave bowl cavities being selectively filled by one cesium ion each. In order to provide insights into the geometrical features and electronic structure of this novel mixed-metal organometallic self-assembly, an in-depth theoretical investigation has been carried out. Specifically, the influence of internal metal binding on the geometry and magnetic coupling of C_20_H_10_˙^3–^ radicals is investigated for Group 1 metals. This study reveals that replacement of the sandwiched potassium ions with larger (Cs) and smaller (Li) ions allows variation of the size of the encapsulated metal belts, and thus enables tuning of the coupling of C_20_H_10_˙^3–^ radicals.

## Abstract 2

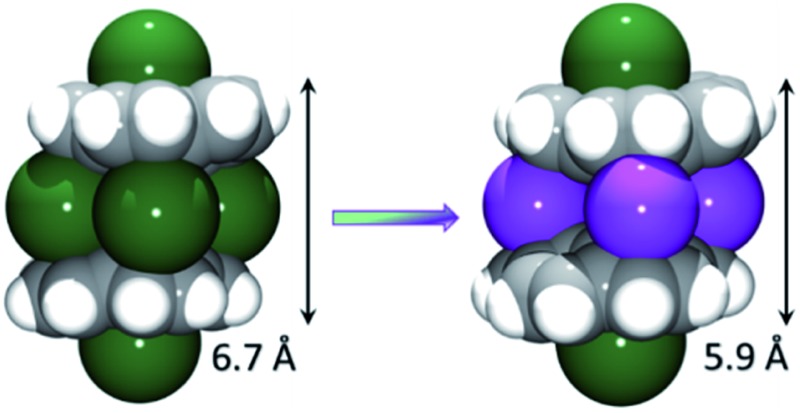
Downsizing of alkali metal belts sandwiched between triply-reduced corannulene decks allows for the fine-tune separation and magnetic coupling of C_20_H_10_˙^3–^ radicals.

## Introduction

Multi-electron reduction of planar and non-planar polycyclic aromatic hydrocarbons (PAHs) has been the focus of broad attention over many years from both fundamental and applied viewpoints.^1–4^ The discoveries of curved and bent π-conjugated molecules, such as fullerenes, nanotubes and their fragments,^5^ further reinvigorated this field with a focus on geometry perturbation, aromaticity, electronic properties, and magnetism of the resulting charged carbon-rich species.^6–9^ Special interest in non-planar radicals with extended π-surfaces^10^ has been driven by their unique magnetic properties^11^ and interesting coupling pathways,^12^ which open up their applications in organic microelectronics and energy storage.^13^ Furthermore, carbon-rich PAHs with pre-designed structures and substitution patterns are used as unique redox-active scaffolds for the generation of biradical^14^ and tetraradicaloid species^15^ and for the preparation of novel metal–organic frameworks.^16^

**Scheme 1:**
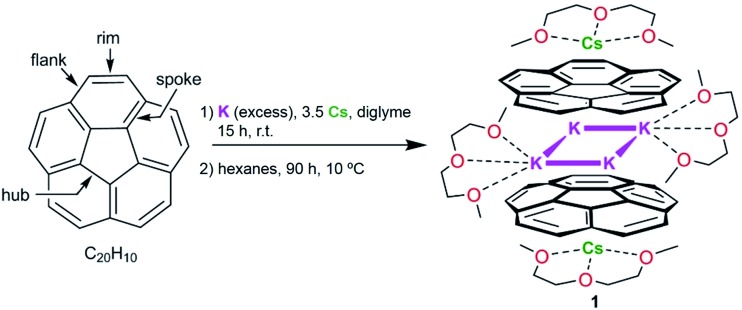
Preparation of **1**.

Our interests focus on bowl-shaped PAHs (often called buckybowls or π-bowls)^17^ which are known to readily uptake multiple electrons in stepwise reduction processes. For example, corannulene (C_20_H_10_, Scheme 1), which maps onto 1/3 of the C_60_-fullerene surface, can acquire up to four electrons, owing to the doubly degenerate nature of its LUMO.^18^ By now, the products of all reduction steps, the mono-,^19^ di-,^19*b*^ tri-,^20^ and tetraanions^21^ of corannulene (C_20_H_10_^*n*–^, *n* = 1–4), have been isolated and crystallographically characterized. These structural studies revealed the remarkable ability of very electron-rich tetrareduced corannulene (which bears one electron per five carbon atoms thus is more electron-rich than the hexaanion of fullerene, C_60_^6–^) to form unique supramolecular products with high numbers of encapsulated alkali metal ions, including unprecedented heterobimetallic combinations.^22^ The binding properties of the transient triply-reduced corannulene remained unknown until our recent report on the first structural characterization of the corannulene trianion isolated with cesium counterions.^20^ X-ray crystallographic analysis revealed that two C_20_H_10_˙^3–^ anions self-assemble into a novel sandwich-type aggregate having four interior and two exterior cesium ions, [Cs^+^//(C_20_H_10_^3–^)/4Cs^+^/(C_20_H_10_^3–^)//Cs^+^]. These structural results^20–22^ clearly differentiate the highly negatively charged tri- and tetra-reduced anions, that show tendencies to self-assemble with multiple metal ions, from mono- and doubly-reduced corannulene, which can be isolated in their “naked” forms.^19,23^ Moreover, the aforementioned homometallic cesium sandwich exhibits several special features, such as a large axial shift of two bowl-shaped decks and the absence of magnetic coupling between two trianion-radicals within the sandwich, as well as an unexpectedly high curvature of corannulene bowls. These experimental observations raised interesting questions about the role of alkali metals in the geometry perturbation of triply-reduced corannulene and its supramolecular aggregation processes. To provide further insights into the unique self-aggregates formed by C_20_H_10_˙^3–^, we set out to investigate the bimetallic reduction of corannulene using K and Cs metals. Herein, we isolated the first mixed-metal product with triply-reduced corannulene and used a combination of experimental and computational techniques to understand its geometric and electronic structures. This task required an in-depth theoretical analysis of the role of encapsulated alkali metal ions, therefore all Group 1 metals ranging in size from Li to Cs were included in the calculations.

## Results and discussion

The first isolation of the corannulene trianion in the form of its crystalline supramolecular product with cesium counterions prompted us to investigate whether other alkali metals are capable of reducing corannulene to the trianion stage and whether these reactions allow its bulk solid-state isolation. Previously, paramagnetic C_20_H_10_˙^3–^ radicals were detected *in situ* by ESR spectroscopy using a reduction reaction of corannulene with lithium metal in THF.^24^ We have observed that Li-induced reduction of corannulene quickly proceeds to the final reduction state, C_20_H_10_^4–^, and thus is impractical for the preparation of products based on transient trianions. However, the outcome of using other Group 1 metals in these reactions remained unclear.

Herein, we found that reduction of C_20_H_10_ in diglyme using an excess of K metal (*ca.* 10 eq.) and a controlled amount of metallic Cs (*ca.* 3.5 eq. in respect to C_20_H_10_) affords a deep-red coloured solution (characteristic of the third reduction step) after 15 hours of stirring at room temperature. Notably, the reduction proceeds significantly faster compared with the cesium-only reaction, requiring more than 60 hours in order to reach the same stage. The UV-vis spectrum of the resulting reaction mixture exhibits a broad absorbance band with *λ*_max_ around 514 nm and a very intense band with *λ*_max_ = 388 nm, which is characteristic of the C_20_H_10_˙^3–^ anion (see ESI, Fig. S1 and S2†). These values are slightly shifted compared to the trianion generated by the Cs-only reduction (*λ*_max_ = 386 nm and 495 nm).

After reaching the third reduction stage, the reaction mixture was filtered to afford a deep-red solution which was layered with hexanes and kept at 10 °C. Dark-red blocks of [K_2_Cs(diglyme)_2_(C_20_H_10_^3–^)] (**1**) were present after 90 hours in *ca.* 50–60% yield (Scheme 1). Crystals of **1** are extremely air- and moisture-sensitive and show very limited solubility in THF.

Notably, further reduction of C_20_H_10_˙^3–^ to the C_20_H_10_^4–^ state was not observed in this work, even when an excess of K and Cs metals was used over an extended reaction time period (one month). It should be mentioned that the reduction of corannulene with potassium metal only, in diglyme, also results in the *in situ* formation of C_20_H_10_˙^3–^ as the major product (Fig. S3†). However, our numerous attempts to crystallize the homometallic potassium analogue of **1** have been unsuccessful so far. In contrast, the crystals of **1** are obtained reproducibly in good yield, thus illustrating the important role of Cs^+^ ions in the formation and crystallization of the title product.

**Fig. 1:**
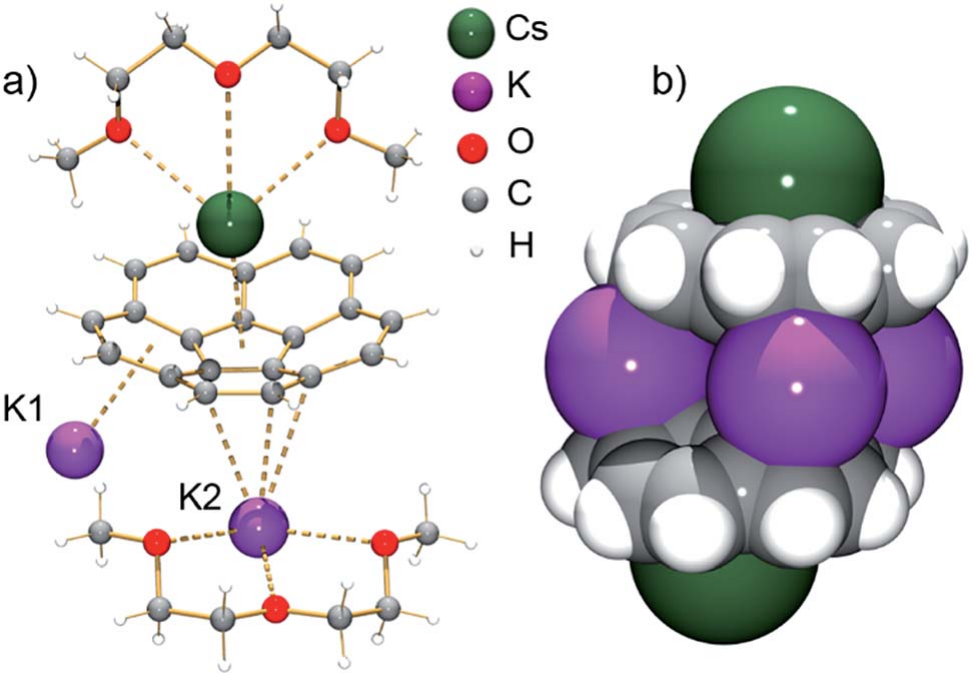
Asymmetric unit (a, ball-and-stick model) and sandwich view (b, space-filling model, diglyme molecules are removed) of **1**.

According to an X-ray diffraction study (see ESI† for more details), the asymmetric unit of **1** consists of one corannulene trianion, two potassium ions, one cesium ion, and two diglyme molecules (Fig. 1a). In the molecular structure, four potassium ions are held between the two triply-reduced corannulene bowls to yield the supramolecular aggregate, [(C_20_H_10_^3–^)/4K^+^/(C_20_H_10_^3–^)]^2–^ (Fig. 1b). The triple-decker sandwich in **1** is similar to that found in the all-cesium product, [(C_20_H_10_^3–^)/4Cs^+^/(C_20_H_10_^3–^)]^2–^. However, four potassium ions are now sandwiched between two C_20_H_10_˙^3–^ anions instead of the larger cesium ions, thus bringing the two corannulene decks notably closer together (5.87 Å *vs.* 6.73 Å). As observed in the cesium analogue, a convex-to-convex arrangement of the two bowls is also found in **1**. The [(C_20_H_10_^3–^)/4K^+^/(C_20_H_10_^3–^)]^2–^ aggregate demonstrates a staggered conformation of two C_20_H_10_˙^3–^ anions that are slipped in respect to each other by 2.093(4) Å (*vs.* 1.843(5) Å in the Cs-only sandwich).

The sandwiched K1, K2, K1′ and K2′ ions form a rectangle with K···K separations of 4.212(4) Å and 5.185(4) Å (compared to those in elemental potassium of 4.54 Å).^25^ Notably, the ionic radius of the K^+^ ion is 1.33 Å *vs.* that of 1.69 Å for Cs^+^.^26^ The K1 ions are bound to both anionic bowls in an η^6^-mode with K1···C interatomic distances of 2.950(3)–3.634(4) Å (K1···C_6(centroid)_: 2.792(3) and 3.041(4) Å). In contrast, the K2 ions are η^3^- and η^6^-bound to the bowls with K2···C distances of 2.945(3)–3.441(3) Å and 2.978(3)–3.354(4) Å (K2···C_6(centroid)_ is 2.818(4) Å). The coordination sphere of the encapsulated K2 ion is completed by chelating diglyme, with the K···O distances ranging from 2.690(3)–2.859(3) Å.

**Fig. 2:**
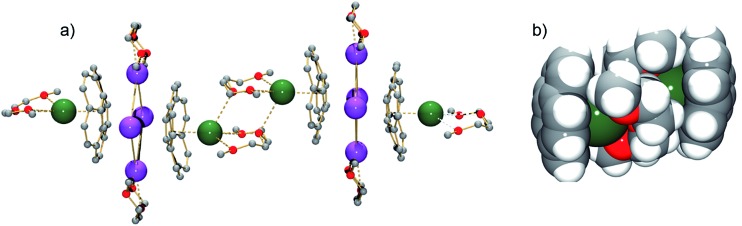
A fragment of the polymeric chain in **1** (a, ball-and-stick, all H-atoms are removed) and a space-filling model showing the [(C_20_H_10_^3–^)/[Cs(diglyme)]_2_/(C_20_H_10_^3–^)]^4–^ connection (b).

As observed with the cesium-generated C_20_H_10_˙^–^ and C_20_H_10_^2–^ anions,^19a,27^ the concave cavities of the charged corannulene bowls are always occupied by large Cs^+^ ions. In **1**, similar to the [Cs^+^//(C_20_H_10_^3–^)/4Cs^+^/(C_20_H_10_^3–^)//Cs^+^] aggregate (**2**), the external concave cavities of the two bowls are filled by one cesium ion each, thus confirming the remarkable selectivity of the Cs^+^ ion towards *endo*-binding, even in the presence of prevalent K^+^ ions. The cesium ion is asymmetrically coordinated to the *endo* surface of C_20_H_10_˙^3–^ in an η^5^-fashion (Cs···C distance of 3.151(3)–3.338(3) Å) with a distance to the center of the five-membered ring of 3.02 Å. The Cs···C distances between the Cs^+^ ion and the benzene rings of C_20_H_10_˙^3–^ are significantly longer (3.374(3)–3.969(4) Å). Additionally, the external cesium ion is bound to three oxygen atoms of diglyme (Cs···O of 3.111(3)–3.469(3) Å) and one oxygen atom of a neighboring diglyme molecule (3.343(3) Å). As a result, the [Cs(diglyme)]^+^ cations serve as building blocks in the formation of a 1D polymer in the crystal structure of **1** (Fig. 2).

**Fig. 3:**
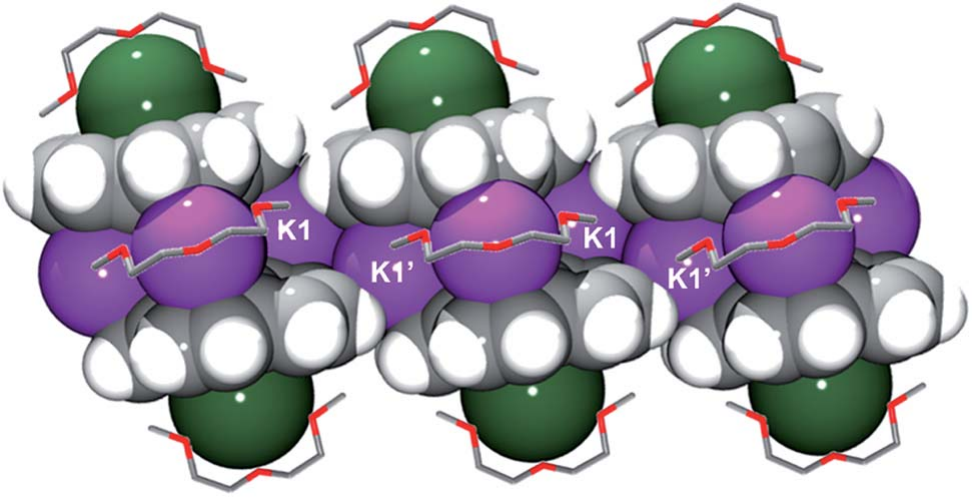
Space filling model of three adjacent sandwiches with the diglyme molecules shown as capped sticks. The hydrogen atoms of diglyme are omitted for clarity.

In the solid state, additional contacts between K1 and the adjacent C_20_H_10_˙^3–^ moieties (3.284(4)–3.430(4) Å) are observed, propagating the structure in the *a*-direction (Fig. 3) and explaining the observed very limited solubility of **1**.

The structural characterization of the mixed-metal sandwich **1** allows its direct comparison with the all-cesium analogue (**2**). Generally, the geometric perturbations of the C_20_H_10_˙^3–^ core in the two products are quite similar. In both cases, the hub C–C bond lengths of C_20_H_10_˙^3–^ are comparable to those in neutral C_20_H_10_ (Table 1),^28^ whereas the spoke and rim C–C bond distances are elongated. The acquisition of three electrons by C_20_H_10_ and the subsequent aggregation with multiple alkali metal ions do not induce any significant flattening of the resulting trianion compared to the parent bowl. The bowl depth of C_20_H_10_˙^3–^ in **1** is 0.808(4) Å *vs.* 0.875(2) Å for neutral C_20_H_10_.^28^ Notably, the curvature of the corannulene trianion separated by the potassium belt in **1** is reduced compared to that in **2** (0.81 *vs.* 0.85 Å), reflecting the greater effect of large encapsulated cesium ions on the bowl depth.

**Table 1:** Key distances (in Å) of C_20_H_10_˙^3–^ in **1** *vs.* corannulene and its cesium analogue (**2**)

	C_20_H_10_^28^	**1**	**2** ^20^
Hub	1.411(2)–1.417(2)	1.403(4)–1.415(5)	1.401(7)–1.429(7)
Spoke	1.376(2)–1.381(2)	1.423(4)–1.430(4)	1.418(8)–1.443(7)
Flank	1.441(2)–1.450(2)	1.416(4)–1.446(4)	1.421(7)–1.439(7)
Rim	1.377(2)–1.387(2)	1.412(4)–1.450(4)	1.416(7)–1.438(8)
Bowl depth	0.875(2)	0.808(4)	0.850(7)

### Magnetic behavior of Cs-only *vs.* K/Cs sandwich products

We have previously found that the sandwich formed by C_20_H_10_˙^3–^ with Cs^+^ ions, [Cs^+^//(C_20_H_10_^3–^)/4Cs^+^/(C_20_H_10_^3–^)//Cs^+^], shows weak antiferromagnetic coupling of the corannulene trianion-radicals through the large alkali metal belt, as confirmed by magnetic measurements and high-level theoretical modeling.^20^ The extreme air- and moisture-sensitivity of the product **2** allowed only a rough estimation of the exchange coupling within the sandwich, with an interaction constant in between –5 and –10 K (with *H* = –2*JS*_1_ *S*_2_) and a gap, *Δ* = |2*J*|, between the singlet ground state and the triplet excited state of about 10–20 K.

Multiple crystalline samples of **1** prepared from different reaction batches have been measured in this work, all showing product decomposition with time even under strictly anaerobic conditions (see ESI and Fig. S5†). Nevertheless, all the magnetic data clearly point to the diamagnetic ground state of the new heterometallic product, [Cs^+^//(C_20_H_10_^3–^)/4K^+^/(C_20_H_10_^3–^)//Cs^+^], and indicate weak antiferromagnetic interaction between two trianion-radicals through the potassium belt in the sandwich. As expected due to a closer proximity of the radicals, this magnetic exchange is slightly greater than in the analogous cesium-only compound, reaching –11.5(5) K (–8.0(3) cm^–1^ and *Δ* = |2*J*| = 16 cm^–1^).

### Computational study

Considering the observed experimental limitations of magnetic measurements caused by the extremely high sensitivity of **1** towards air and moisture, we turned to theoretical investigations. Previously, we have demonstrated that the PBE0/def2-TZVP+ECP(K,Cs)//cc-pVDZ(C,H,O) level of theory provides an adequate balance between accuracy and computational effort (see details in ESI†). Similar to the Cs-only system,^20^ four different models were considered initially: (i) the simplest fully optimized [Cs^+^//(C_20_H_10_^3–^)/4K^+^/(C_20_H_10_^3–^)//Cs^+^] model (**1**-K-small); (ii) the same model, but with the core structure taken from the X-ray experiment and kept unchanged, while the positions of the hydrogen atoms were optimized (**1H**-K-small); (iii) the fully optimized model, in which all solvent molecules were considered explicitly (**1**-K-full); (iv) the same model as **1**-K-full, but with only the hydrogen atom positions optimized (**1H**-K-full), whereas the rest was taken from the crystal structure and kept frozen (see further details in ESI†).

#### Geometry and electronic structure

As expected, optimization of the most accurate **1**-K-full model in its triplet state resulted in the closest resemblance of its geometrical parameters (Table 2) with those observed in the experimental X-ray crystal structure of **1** (Table 1). For instance, the calculated axial shift (or sliding, see Table 2 scheme) of the two bowls within the sandwich is reproduced with better accuracy in the case of **1**-K-full (1.51 Å) than in the simplest **1**-K-small model (0.84 Å). This clearly shows the high sensitivity of the bowl shift with respect to its external environment and might be indicative of relatively weak coupling between two C_20_H_10_˙^3–^ radicals. At the same time, the bowl depth was found to be similar in the **1**-K-small and **1**-K-full models (0.768 Å and 0.758 Å, respectively). The calculated distances between the bowls (*d*_1_ and *d*_2_ parameters in Table 2) also do not differ much for the two selected models. Importantly, all the major calculated geometrical parameters closely resemble the experimental X-ray crystal data of **1**, as was also seen for the Cs-only product.^20^

**Table 2:** Selected geometrical and magnetic parameters for the **1**-M-small (where M = Li, Na, K, Rb, Cs), **1H**-K-small/full and **1**-K-full models, calculated at the PBE0/def2-TZVP(+ECP)(metal)//cc-pVDZ(C,H) level of theory

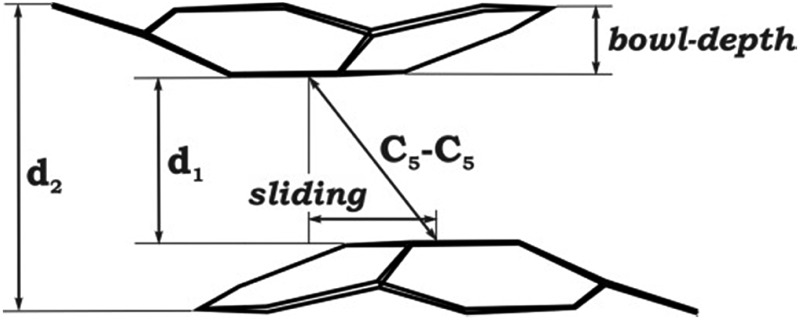								
Parameter	Model							
	**1**-M-small					**1H**-K-small	**1H**-K-full	**1**-K-full
	M = Li	M = Na	M = K	M = Rb	M = Cs			
Bowl-depth	0.510	0.731	0.768	0.814	0.826	0.808	0.808	0.758
*d*_1_	3.180	3.723	4.348	4.629	4.865	4.247	4.247	4.417
*d*_2_	4.252	5.204	5.952	6.283	6.541	5.872	5.872	5.967
Bowl sliding	1.601	1.171	0.840	0.750	0.453	2.091	2.091	1.510
C_5_–C_5_	3.560	3.904	4.430	4.691	4.891	4.735	4.735	4.675
M···M (min)	3.576	3.844	4.265	4.481	4.668	4.212	4.212	4.340
M···M (max)	4.516	4.210	4.468	4.630	4.804	5.185	5.185	4.701
*Δ*(M···M)	0.940	0.366	0.203	0.149	0.136	0.973	0.973	0.361
2*J* ^*a*^	+32.32	–3.00	–6.44	–24.99	–2.39	+1.50	–0.69	–2.62

^*a*^The *J*-coupling constant (in cm^–1^) is calculated using the Yamaguchi formula (broken-symmetry BS(1,1) solution)^29^ at the PBE0/TZVP/ZORA level of theory as implemented in the ORCA program suite.^30^

Previously, our calculations revealed that the large Cs^+^ ions jammed between bowl-shaped triply-reduced corannulene anions prevent any significant magnetic coupling in the sandwich-like aggregates.^20^ The electronic structure of such an assembly was found to be best described as a system with two uncoupled radicals. Replacing Cs^+^ ions with much smaller K^+^ ions gave a 2*J*-coupling constant of –0.69 cm^–1^ for the **1H**-K-full model calculated using a broken-symmetry approach (see details in ESI†). Relaxation of the geometry during the optimization procedure led to a slight increase in the anti-ferromagnetic coupling between the trianion-radicals (2*J* = –2.62 cm^–1^) in the **1**-K-full system. Interestingly, the removal of coordinated solvent molecules from the full models showed some influence on the magnetic coupling. The corresponding 2*J*-constants were computed to be +1.50 cm^–1^ and –6.44 cm^–1^ for the **1H**-K-small and **1**-K-small models, respectively, thus illustrating that at this level of theory it is difficult to make unambiguous conclusions about the ground state and magnetic coupling in the target systems. Therefore, we turned to the highly accurate multireference Møller–Plesset perturbation theory of the second order (MRMP2).^31^ Since the application of this technique to the full systems is not feasible, we mainly focused on using the **1H**-K-small and **1**-K-small models. The active space for the reference CASSCF wavefunction included 14 electrons over 8 orbitals, CASSCF(14,8). Calculations performed at the MRMP2 level of theory revealed that the energy gap, *Δ*, between the open-shell singlet and triplet states is equal to –6.46 cm^–1^ for **1H**-K-small and –54.43 cm^–1^ for **1**-K-small. Notably, a good agreement between the *J*-constant values for the **1H**-K-small model and the experimentally measured one (–8.0 cm^–1^) was observed. There is also an agreement with the experiment-based conclusion that the anti-ferromagnetic coupling in **1** is stronger than that in **2** (–0.01 cm^–1^ calculated at the same level of theory).^20^ Relaxation of the geometry increased the magnitude of the coupling as it is exemplified by the **1**-K-small model. The same trend was observed in the full models, for which the coupling parameters were calculated at the BS-DFT level of theory. This clearly shows the applicability of the broken-symmetry DFT approach to the modeling of magnetic coupling in such complicated systems. Although it showed some deficiency in providing the exact numbers, the qualitative trends are reproduced correctly.

**Fig. 4:**
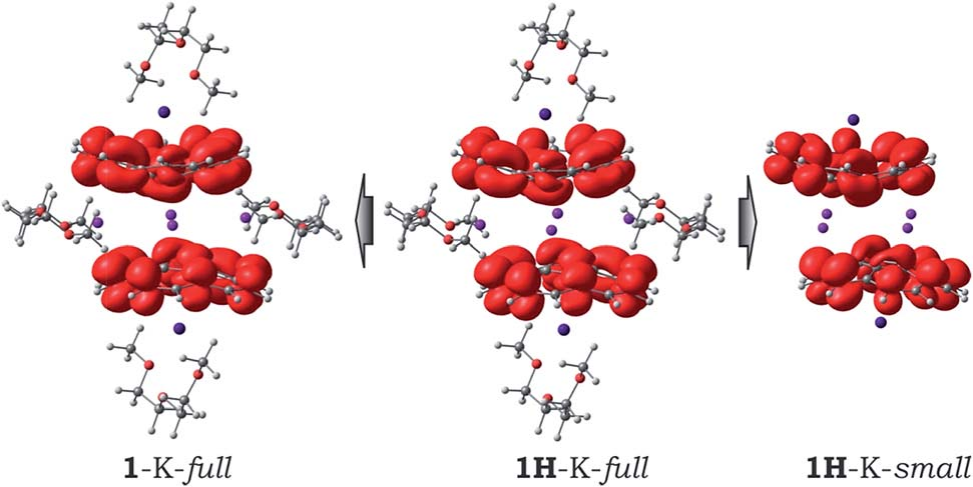
Spin density distribution in the **1H**-K-full, **1**-K-full, and **1H**-K-small models.

Interestingly, due to weak magnetic coupling between the bowl-shaped fragments, the charge and spin distributions in the sandwich systems remain similar to those of the isolated C_20_H_10_˙^3–^ species (see ESI† for details). Only a slight polarization of charge distribution was found in the **1H**-K-small model due to the axial shift of the bowls with respect to each other. The spin density of the unpaired electrons is exclusively localized on the negatively-charged corannulene bowls. Moreover, the topology of the spin density is not notably influenced by the presence of solvent molecules and/or by crystal packing effects (Fig. 4).

#### The effect of alkali metal size

**Fig. 5:**
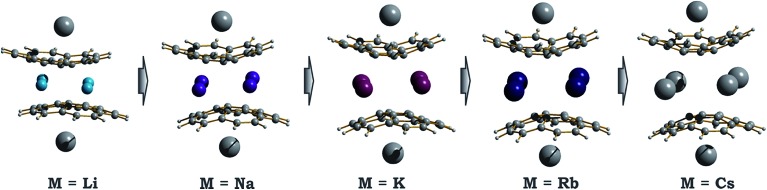
Equilibrium geometry configurations for **1**-M-small systems, where M = Li, Na, K, Rb and Cs (PBE0/def2-TZVP(M)//cc-pVDZ).

Since the direct comparison of **1** and **2** clearly revealed the influence of alkali metal belts sandwiched between the triply-reduced corannulene bowls on the geometry and magnetic coupling of the resulting products, we turned to an in-depth analysis of this effect by taking other Group 1 metal ions (Li^+^, Na^+^ and Rb^+^) into consideration. In this part, only the fully relaxed small models, **1**-M-small (M = Li, Na, K, Rb, and Cs), were considered. We have previously shown computationally that replacement of the external concave bound cesium ions with much smaller lithium ions to form the [Li^+^//(C_20_H_10_^3–^)/4Cs^+^/(C_20_H_10_^3–^)//Li^+^] aggregate results in the flattening of the bowl depth to 0.775 Å (*vs.* 0.850(7) Å in the Cs-only analogue).^20^ In this work, the concave cavities in all of the calculated sandwich aggregates were filled by Cs^+^ ions to allow the clear evaluation of the role of encapsulated alkali metals. The resulting geometrical configurations for the series of [Cs^+^//(C_20_H_10_^3–^)/4M^+^/(C_20_H_10_^3–^)//Cs^+^] products are depicted in Fig. 5 with selected parameters collected in Table 2.

As expected, the downsizing of the positively-charged tetranuclear alkali metal belts held between the C_20_H_10_˙^3–^ bowls results in the decreasing of the supramolecular aggregate height, as can be illustrated by the *d*_2_ parameter (Table 2). The distance between the two corannulene bowls (*d*_1_) also decreases from 4.417 Å to 3.180 Å going from Cs to Li, which may result in the increased coupling between the C_20_H_10_˙^3–^ radicals. In addition, the effect of the alkali metal size on the curvature of the bowl-shaped decks is observed. The calculated bowl-depth of C_20_H_10_˙^3–^ increases when going from the **1**-Li-small (0.510 Å) to **1**-Cs-small (0.826 Å) system. This variation shows the remarkable flexibility of the corannulene framework and provides an argument that its curvature in the sandwich-like aggregates depends not only on the charge state (as predicted earlier^32^) but, importantly, on the nature and size of the encapsulated metal ions.

Interestingly, decreasing the M^+^ ion size in the calculated systems also led to a pronounced shift of one bowl with respect to another (Table 2). The largest bowl slip was computed for the **1**-Li-small system (1.601 Å), whereas the smallest one was found for its Cs-derivative (0.453 Å). Previously, such a shift was attributed exclusively to the influence of coordinated solvent molecules and crystal packing effects. Herein, the calculated shifts for all **1**-M-small systems unambiguously reveal that the size and nature of the alkali metals, constituting the positively charged belts, play a very important role. As a consequence, the shape of the encapsulated tetranuclear metal unit undergoes a smooth transformation from almost square-planar in the **1**-Cs-small system (*Δ*(M···M) = 0.136 Å) to a very pronounced rectangular shape in the **1**-Li-small system (*Δ*(M···M) = 0.940 Å).

**Fig. 6:**
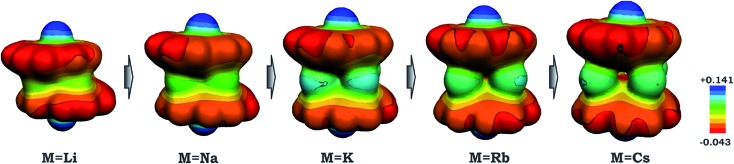
Molecular electrostatic potential maps for **1**-M-small systems, where M = Li, Na, K, Rb and Cs (PBE0/def2-TZVP(M)//cc-pVDZ).

As a result of this bowl sliding clearly affecting the product geometry, one can detect a noticeable polarization in the charge distribution along the series of sandwich-like aggregates (as exemplified by the molecular electrostatic potential (MEP) maps in Fig. 6). While the charge state of the corannulene fragments remains –3, the negative charge on their π-surface is becoming more and more localized in order to minimize the electrostatic repulsion between the bowls. For instance, in the case of the **1**-Cs-small system having the greatest separation between the two bowls (Table 2) and minimal sliding, the charge distribution is almost symmetrical. At the same time, for the **1**-Li-small system (with the smallest *d*_1_ and the largest axial shift between the bowls), the localization of negative charges was found to be the most pronounced in the series.

**Fig. 7:**
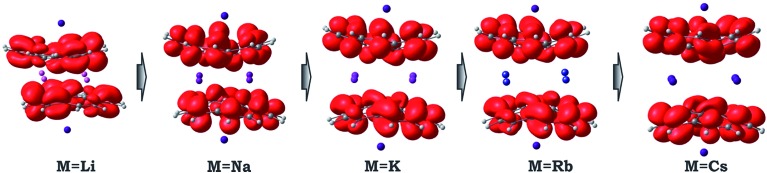
Spin density distribution in **1**-M-small systems, where M = Li, Na, K, Rb and Cs (PBE0/def2-TZVP(M)//cc-pVDZ).

Considering the significant variations in geometry when going from Cs to Li (Table 2), one may expect accompanied changes in the spin density distribution and, consequently, in the magnetic coupling of the two C_20_H_10_˙^3–^ radicals. The increased shift of the corannulene bowls with respect to each other (parameter “bowl sliding” in Table 2) notably stabilizes the localization of the unpaired electrons on opposite sides of the supramolecular aggregate, as exemplified by the spin density distribution (Fig. 7). This localization is almost negligible in the **1**-Cs-small system but reaches a maximum in the **1**-Li-small system. As expected, the associated stabilization results in an increasing ferromagnetic interaction between the bowl-shaped radicals and in a decreasing anti-ferromagnetic component. This can be clearly illustrated by comparison of the **1H**-K-small (sliding = 2.09 Å) and **1**-K-small (sliding = 0.840 Å) systems, for which the magnetic coupling constants were calculated to be 2*J* = +1.50 cm^–1^ and –6.44 cm^–1^ (at the BS-DFT level of theory), respectively. The same trend was observed for the **1H**-Cs-small and **1**-Cs-small models (sliding = 1.84 Å and 0.453 Å; *J* = +3.84 cm^–1^ and –2.39 cm^–1^, respectively).^20^ Eventually, this ferromagnetic interaction is maximized in the case of the **1**-Li-small system (2*J* = +32.32 cm^–1^), where the axial sliding is the most pronounced (1.601 Å).

At the same time, another tendency in the magnetic coupling between the curved corannulene radicals as a function of the product geometry was also observed, namely an increase in anti-ferromagnetic coupling with decreasing distance *d*_1_ (Table 2). In order to provide further evidence for this trend, theoretical modeling of the modified **1**-Li-small model (hereafter called **1**-Li-small-iso) was performed at the same level of theory. In this new model, two bowl fragments were placed on top of each other in a non-sliding fashion, whereas the distance *d*_1_ was kept exactly the same as in the fully relaxed **1**-Li-small system (see details in ESI†). Thus, the influence of the bowl shift on the magnetic coupling between the corannulene radicals was excluded. The 2*J*-constant for the **1**-Li-small-iso model was calculated to be –42.50 cm^–1^ (in contrast to 2*J* = +32.32 cm^–1^ calculated for the **1**-Li-small model), which clearly illustrates the strengthening of an anti-ferromagnetic component.

Hence, the actual magnetic coupling between two corannulene radicals is a result of the interplay between these two trends in the product geometry change induced by the downsizing of the encapsulated alkali metal belt from the largest Cs^+^ ions to the smallest Li^+^ ions. As one might expect for the two opposing trends, the correlation between alkali metal size and magnetic coupling is not linear and may have a maximum/minimum. This is what was observed in the case of M = Rb, where the anti-ferromagnetic coupling was found to be maximized.

In order to complete the description of the magnetic coupling for the series, an analysis of the interactions between the bowl-shaped corannulene fragments and the positively-charged alkali metal belt was performed using the Energy Decomposition Analysis (EDA) approach. The accepted fragmentation scheme was the same as that proposed in a recent study.^20^ Three interacting fragments were considered (Fig. S11†), namely two [(Cs)(C_20_H_10_)]^2–^ and one [M_4_]^4+^ species (M = Li, Na, K, Rb and Cs). Such fragmentation allows one to tentatively evaluate the interactions in the supramolecular aggregates as well as to estimate the coupling between the negatively-charged bowls. The results of the EDA analysis are collected in Table 3.

**Table 3:** Results of the EDA analysis for **1**-M-small (where M = Li, Na, K, Rb and Cs) models, calculated at the PBE0/TZ2P/ZORA level of theory

Parameter	Compound				
	**1**-Li-small	**1**-Na-small	**1**-K-small	**1**-Rb-small	**1**-Cs-small
Δ*E*_int_	–1230.57	–1133.72	–1024.70	–981.60	–942.57
Δ*E*_orb_	–383.30	–298.56	–256.84	–239.17	–241.89
Δ*E*_elstat_	–977.79	–946.44	–892.07	–867.31	–853.45
Δ*E*_Pauli_	+130.52	+111.27	+124.22	+124.88	+152.77

As shown in Table 3, the coupling between corannulene bowls gets significantly stronger when going from Cs to Li, as exemplified by the total interaction energy Δ*E*_int_ (which changes from –1230.57 kcal mol^–1^ to –942.57 kcal mol^–1^, respectively). This finding is in excellent agreement with previous conclusions, based on charge and spin density distributions as well as on magnetic coupling in the target systems. Importantly, both of the attractive components of Δ*E*_int_, namely the orbital (Δ*E*_orb_) and electrostatic (Δ*E*_elstat_) components, follow exactly the same trends. At the same time, the repulsive Pauli interaction (Δ*E*_Pauli_) shows an opposite tendency in the series, again making systems with small alkali metal ions sandwiched between the corannulene bowls more stable. However, even the high stability of the **1**-Li-small system with triply-reduced bowls does not make it as stable as the system based on tetrareduced corannulene. The total interaction energy in such a **1^4–^**-small system was previously calculated to be –1536.03 kcal mol^–1^.^20^ The main difference should arise from the dramatically stronger electrostatic component in the latter (–977.79 kcal mol^–1^ in the **1**-Li-small system *vs.* –1452.97 kcal mol^–1^ in the **1^4–^**-small system).

## Conclusions

In this work, the isolation and X-ray structural characterization of the first heterobimetallic sandwich-type aggregate formed by triply-reduced corannulene, [Cs^+^//(C_20_H_10_^3–^)/4K^+^/(C_20_H_10_^3–^)//Cs^+^], has been accomplished. The successful synthesis of this mixed-metal assembly based on the selectivity of the concave and convex binding of the corannulene trianion towards potassium and cesium ions provides a remarkable experimental illustration of the fascinating coordinating properties of highly charged π bowls.

Furthermore, analysis of the structural and magnetic data for the title product has opened up the first discussion on the role of encapsulated metals on the perturbation of molecular geometry and magnetic coupling of bowl-shaped radicals within sandwich-type architectures. The variation of tetranuclear alkali metal belts from Li to Cs in the [Cs^+^//(C_20_H_10_^3–^)/4M^+^/(C_20_H_10_^3–^)//Cs^+^] aggregates revealed unique structural and electronic transformations within the series. Remarkably, the replacement of the sandwiched K ions with larger (Cs and Rb) and smaller (Li) ions is accompanied by notable geometrical changes of the sandwich architectures, as reflected by the different axial shifts and separations of the corannulene trianion radicals. Notably, the downsizing of the sandwiched alkali metal belts allows for the tuning of the coupling of C_20_H_10_˙^3–^ radicals from anti-ferromagnetic to ferromagnetic in nature.
