# Supplementary material for: Tuning the separation and coupling of corannulene trianion-radicals through sizable alkali metal belts
Source: Chem Sci. 2017 Feb 23;8(4):3137–45. doi: 10.1039/c6sc05370j (PMC5413885; doi:10.1039/c6sc05370j)
Supplement: Supplementary file 1 [file SC-008-C6SC05370J-s001.pdf]

Electronic Supplementary Material (ESI) for Chemical Science.
This journal is © The Royal Society of Chemistry 2017

Supporting Information

Tuning Separation and Coupling of Corannulene Trianion-Radicals through Sizable Alkali Metal Belts

Sarah N. Spisak,[a] Andrey Yu. Rogachev,[b,*] Alexander V. Zabula,[a,c] Alexander S. Filatov,[a] Rodolphe Clérac,[d,e] Marina A. Petrukhina[a,*]

[a] *Department of Chemistry, University at Albany, State University of New York, Albany, NY 12222, USA. E-mail: mpetrukhina@albany.edu*

[b] *Department of Chemistry, Illinois Institute of Technology, Chicago, IL 60616, USA. E-mail: arogache@iit.edu*

[c] *Department of Chemistry, University of Pennsylvania, Philadelphia, PA 19104, USA*

[d] *CNRS, CRPP, UPR 8641, F-33600 Pessac, France*

[e] *Univ. Bordeaux, CRPP, UPR 8641, F-33600 Pessac, France*

I. Experimental Details S3

Materials and Methods S3

Preparation of [K_2Cs(diglyme)$_2$][$C_{20}H_{10}^{3-}$] (1) S3

Figure S1. UV-vis spectra of [$C_{20}H_{10}^{3-}$/4Cs^+/$C_{20}H_{10}^{3-}$]$^{2-}$ and [$C_{20}H_{10}^{3-}$/4K^+/$C_{20}H_{10}^{3-}$]$^{2-}$ S4

Figure S2. UV-vis spectra of [$C_{20}H_{10}^{3-}$/4Cs^+/$C_{20}H_{10}^{3-}$]$^{2-}$ and crystals of **1** S4

Figure S3. UV-vis spectra of kinetic measurement of K/$C_{20}H_{10}$ in diglyme S4

Figure S4. Variable-temperature ^{1}H NMR spectra of **1** S5

II. Magnetic Measurements S5

Figure S5. Temperature dependence of the χT product at 0.1 T of **1** S6

III. Crystal Structure Determinations and Refinement of 1 S7

Table S1. Crystallographic Data for **1** S8

Figure S6. ORTEP drawing of the asymmetric unit of **1** with thermal ellipsoids shown at the 40% probability level S9

IV. Calculation details S10

Figure S7. Equilibrium structures for models **1**-K-*small*, **1H**-K-*small*, and **1**-K-*full* S11

Figure S8. Equilibrium geometry configurations for **1**-M-*small* systems (M = Li–Cs) S11

Figure S9. CASSCF(14,8) natural orbitals along with occupancies for **1H**-K-*small* model S12

Figure S10. CASSCF(14,8) natural orbitals along with occupancies for **1**-K-*small* model S13

Table S2. Cartesian coordinates for **1-K-***full* system, optimized at the PBE0/def2-TZVP(K,Cs)//cc-pVDZ(C,H,O) level of theory S13

Table S3. Cartesian coordinates for **1H-K-***full* system, optimized at the PBE0/TZVP/ZORA level of theory S16

Table S4. Cartesian coordinates for **1H-K-***small* system, optimized at the PBE0/TZVP/ZORA level of theory S18

Table S5. Cartesian coordinates for **1**-Li-*small* system, optimized at the PBE0/def2-TZVP(Li,Cs)//cc-pVDZ(C,H,O) level of theory S19

Table S6. Cartesian coordinates for **1**-Na-*small* system, optimized at the PBE0/def2-TZVP(Na,Cs)//cc-pVDZ(C,H,O) level of theory S20

Table S7. Cartesian coordinates for **1**-K-*small* system, optimized at the PBE0/def2-TZVP(K,Cs)//cc-pVDZ(C,H,O) level of theory S21

Table S8. Cartesian coordinates for **1**-Rb-*small* system, optimized at the PBE0/def2-TZVP(Rb,Cs)//cc-pVDZ(C,H,O) level of theory S22

Table S9. Cartesian coordinates for **1**-Cs-*small* system, optimized at the PBE0/def2-TZVP(,Cs)//cc-pVDZ(C,H,O) level of theory S23

Table S10. NBO charges in **1**-K-*full* system, optimized at the PBE0/def2-TZVP(K,Cs)//cc-pVDZ(C,H,O) level of theory S24

Table S11. NBO charges in **1H**-K-*full* system, optimized at the PBE0/TZVP/ZORA level of theory S27

Table S12. NBO charges in **1H**-K-*small* system, optimized at the PBE0/TZVP/ZORA level of theory S29

Table S13. NBO charges in **1**-Li-*small* system, optimized at the PBE0/def2-TZVP(Li,Cs)//cc-pVDZ(C,H,O) level of theory S30

Table S14. NBO charges in **1**-Na-*small* system, optimized at the PBE0/def2-TZVP(Na,Cs)//cc-pVDZ(C,H,O) level of theory S31

Table S15. NBO charges in **1**-K-*small* system, optimized at the PBE0/def2-TZVP(K,Cs)//cc-pVDZ(C,H,O) level of theory S32

Table S16. NBO charges in **1**-Rb-*small* system, optimized at the PBE0/def2-TZVP(Rb,Cs)//cc-pVDZ(C,H,O) level of theory S34

Table S17. NBO charges in **1**-Cs-*small* system, optimized at the PBE0/def2-TZVP(Cs)//cc-pVDZ(C,H,O) level of theory S35

Table S18. Absolute energies of all systems PBE0/def2-TZVP(*metal*)//cc-pVDZ(C,H,O) level of theory S36

EDA analysis of 1-M-small systems S36

Figure S11. EDA fragmentation scheme in **1**-M-*small* models, where M = Li–Cs S37

Table S19. Results of modelling of magnetic coupling (2*J*, in cm^{-1}) in **1**-M-*small* and **1H**-M-*small* systems (M = K, Cs) at different levels of theory S37

V. References S38

I. Experimental Details

Materials and Methods. All manipulations were carried out using break-and-seal[1] and glove-box techniques under an atmosphere of argon. Hexanes were dried over Na/benzophenone and distilled prior to use. Diglyme and THF-d_8 were dried over NaK_2 alloy and vacuum-transferred. Alkali metals were purchased from Strem Chemicals. Corannulene was prepared as described previously[2] and sublimed at 175 °C prior to use. The UV-vis spectra were recorded on a PerkinElmer Lambda 35 spectrometer. The ^{1}H NMR spectra were measured on a Bruker AC-400 spectrometer at 400 MHz and were referenced to the resonances of the corresponding solvent used. Elemental analysis was performed by Complete Analysis Laboratories, Ins., Parsippany, NJ.

Preparation of $[K_2Cs(diglyme)_2][C_{20}H_{10}^{3-}]$ (1)

Diglyme (3 mL) was added to a flask containing Cs (19 mg, 0.14 mmol), excess K (*ca*. 10 eq.), and corannulene (10 mg, 0.04 mmol). The resulting deep green solution was stirred at room temperature for 15 h affording a deep red mixture. This mixture was filtered; the red filtrate was layered with hexanes (2.5 mL) and kept at 10 °C. Dark red blocks of **1** were present in 90 h. The solution was decanted, and the crystals were washed several times with hexanes and dried *in vacuo*. Yield: 32 mg, 60%. UV-vis (diglyme, nm): λ_{max} = 388, 514. Anal. Calcd for $C_{32}H_{38}K_2CsO_6$: C, 52.67; H, 5.25; Found: C, 52.78 ; H, 5.24. ^{1}H NMR (400 MHz, THF-d_8, 20 °C, ppm): δ = 3.32, 3.48, and 3.58. ^{1}H NMR (400 MHz, THF-d_8, −60 °C, ppm): δ = 3.35 (OCH_3), 3.51 (CH_2), and 3.59 (CH_2). ^{1}H NMR signals of free diglyme in THF-d_8 are 3.28 ppm, 3.43 ppm, and 3.53 ppm.

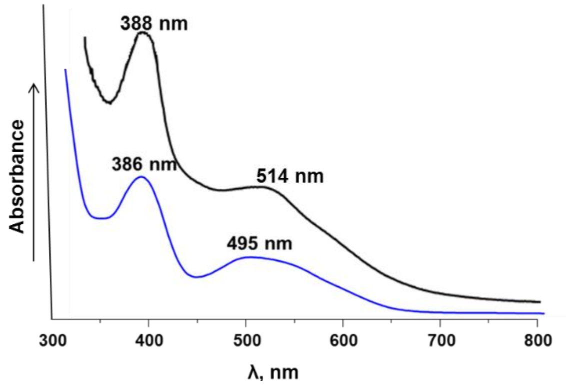

Figure S1. UV-vis spectra of $[C_{20}H_{10}^{3-}/4Cs^{+}/C_{20}H_{10}^{3-}]^{2-}$ (blue) and *in-situ* generated $[C_{20}H_{10}^{3-}/4K^{+}/C_{20}H_{10}^{3-}]^{2-}$ (black).

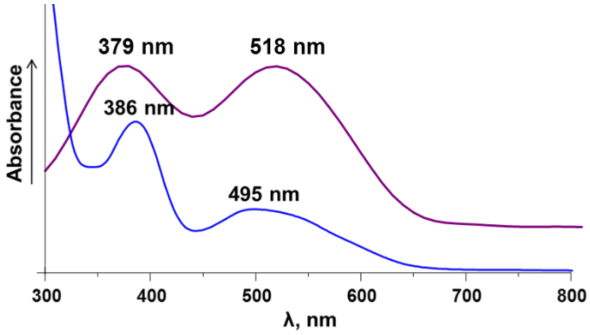

Figure S2. UV-vis spectra of $[C_{20}H_{10}^{3-}/4Cs^{+}/C_{20}H_{10}^{3-}]^{2-}$ (blue) and crystals of **1** dissolved in diglyme (purple).

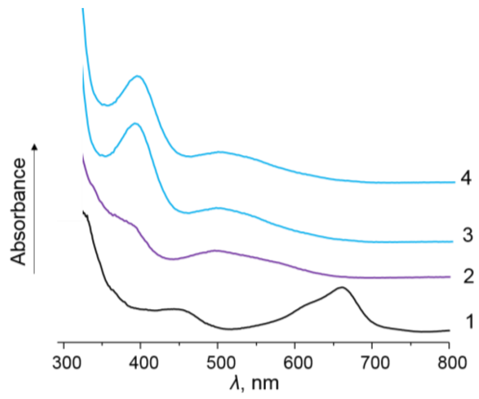

**Label**	**Time**
1	5 minutes
2	1 hour
3	15 hours
4	5 days

Figure S3. UV-vis spectra of kinetic measurement of $K/C_{20}H_{10}$ in diglyme. Monoanion (black): 445 nm and 659 nm. Dianion (purple): 387(sh) nm and 494 nm. Trianion (blue): 390 nm and 496 nm.

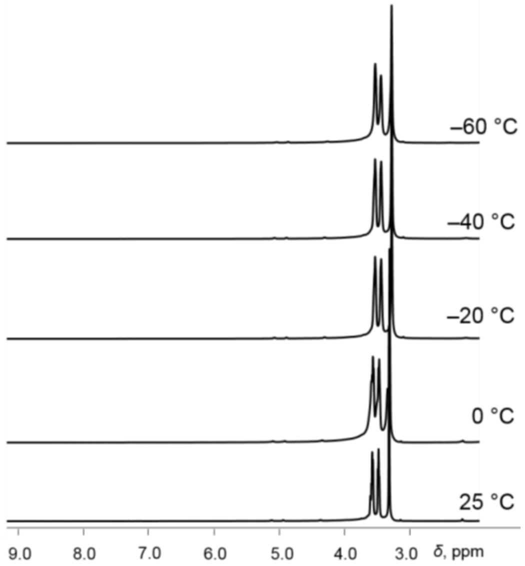

Figure S4. Variable-temperature ^{1}H NMR spectra of **1**. ^{1}H NMR signals of free diglyme in THF-d_8 are 3.28, 3.43, and 3.53 ppm.

II Magnetic Measurements

Magnetic measurements were carried out with the use of Quantum Design MPMS-XL SQUID magnetometer. This instrument works between 1.8 and 400 K with applied dc fields ranging from –7 to 7 T. Measurements were performed on polycrystalline samples of **1** (9.8, 9.5, 10.1, 15.8 and 19.6 mg) and **2** (10.2 and 19.7 mg) manipulated in a drybox under nitrogen atmosphere and sealed in a polypropylene bag (3 × 0.5 × 0.02 cm; typically 15 to 30 mg). Prior to the experiments, the field-dependent magnetization was measured at 100 K in order to confirm the absence of any bulk ferromagnetic impurities. The magnetic data were corrected for the sample holder and the intrinsic diamagnetic contributions.

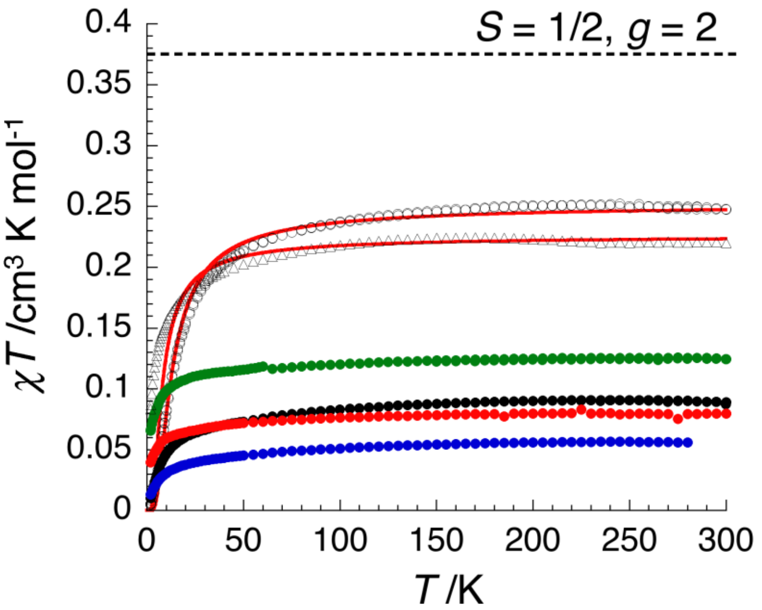

Figure S5. Temperature dependence of the χT product at 0.1 T (χ is defined as magnetic susceptibility equal to M/H per mole of **1** or **2**, ie. per one mole of corannulene trianion-radical) for five different polycrystalline samples of **1** (circles) and one sample of **2** (triangles). The red solid lines are the best fit of the experimental data to the $S = ½$ Heisenberg dimer model.[3]

Five polycrystalline samples of **1** and **2** prepared from different reaction batches have been measured over the period of one year (with several days delay between the preparation and measurements due to sample shipments). All our attempts to obtain a set of magnetic data in agreement with the presence of an $S = ½$ spin (doted line in Figure S5; $C = 0.375$ cm^{3}K/mol with $g = 2$) have failed likely due to extreme air- and moisture sensitivity of the compound as shown in Figure S5. Nevertheless, the vanishing of the χT product at low temperature is a clear indication of an antiferromagnetic interaction between two corannulene radicals and indicates the diamagnetic ground state of the sandwich-type product. By increasing the temperature, the triplet

excited state (S = 1) is thermally populated inducing an increase of the magnetic susceptibility. The theoretical expression for the magnetic susceptibility of an antiferromagnetic coupled S = 1/2 dimer is well represented by the Bleaney - Bowers model[3]

$$c = \frac{Ng^2 m_B^2}{k_B T\left(3 + \exp\left(-2J / k_B T\right)\right)}$$

where N, μ_B and k_B, have their usual meanings, g is the Landé factor of the corannulene radical, and J is the magnetic exchange constant between paramagnetic corannulene radicals (S = 1/2) in the spin dimer ($\boldsymbol{H} = -2J\boldsymbol{S}_1 \bullet \boldsymbol{S}_2$; Please note that the above expression of the susceptibility is normalized per radical). An excellent agreement with the experimental data is found with this model which yields J/k_B = - 7.1 (8) K (–4.9(6) cm^{-1}) for **2** and J/k_B = –11.5 (5) K (–8.0(3) cm^{-1}) for **1** (solid lines in Figure S5; For **1**, the found Curie constant is 0.25 cm^3K/mol, showing that the radicals are preserved from decomposition at best around 67% in one of the five samples).

III. Crystal Structure Determinations and Refinement of 1

Data collection was performed on a Bruker SMART APEX CCD-based X-ray diffractometer with graphite-monochromated Mo-Kα radiation (λ = 0.71073 Å) at T = 100(2) K. Data were corrected for absorption effects using the empirical method SADABS.[4] The structure was solved by direct methods and refined by a full-matrix least-squares procedure using OLEX2[5] (XL refinement program version 2014/7).[6] All elements including atoms of the disordered {Cs(diglyme)} moiety were refined with anisotropic thermal parameters. Disorder was modelled with the application of soft geometric (SADI) restraints. EADP and RIGU were utilized on thermal parameters. The ratio of two parts refined to about 60:40. Cs atom was included into the modelling. Hydrogen atoms were included at idealized positions using the riding model. Crystal

contained some disordered solvent molecules that could not be modelled. The diffuse contribution to scattering was treated by application of the program SQUEZZE[7] as implemented in Platon[8] using the "fab" file construct. This construct allows the solvent density distribution to be added to calculation of structure factors rather than modifying the observed intensities through the subtraction of a solvent contribution. SQUEEZE algorithm located a void, centered at (0, 0.5, 0.5), with a volume of 264 Å^3 and the electron count of 50. This can account for a partially occupied molecule of hexanes. For further crystal and data collection details see Table S1.

Table S1. Crystallographic Data of **1**.

Empirical formula	$C_{32}H_{38}CsK_2O_6$
Formula weight	729.73
Temperature/K	100.0
Crystal system	triclinic
Space group	P-1
a/Å	9.8938(9)
b/Å	13.3712(12)
c/Å	15.5210(14)
α/°	64.4920(10)
β/°	78.4840(10)
γ/°	71.7550(10)
Volume/Å^3	1755.0(3)
Z	2
ρ_{calc}, g/cm^3	1.381
μ/mm^{-1}	1.330
F(000)	742.0
Crystal size/mm^3	0.48 × 0.32 × 0.31
Radiation	MoK$_\alpha$ (λ = 0.71073)
2Θ range for data collection/°	3.492 to 56.482
Index ranges	$-13 \leq h \leq 13$, $-17 \leq k \leq 17$, $-20 \leq l \leq 20$
Reflections collected	15218

Independent reflections	7805 [R_{int} = 0.0178, R_{sigma} = 0.0258]
Data/restraints/parameters	7805/105/413
Goodness-of-fit on F^2	1.048
Final R indexes [$I \geq 2\sigma(I)$]	R_1 = 0.0497, wR_2 = 0.1158
Final R indexes [all data]	R_1 = 0.0549, wR_2 = 0.1204
Largest diff. peak/hole / e·Å^{-3}	1.64/–2.94

[a] $R_1 = \Sigma||F_o|-|F_c||/\Sigma|F_o|$. [b] $wR_2 = [\Sigma[w(F_o^2-F_c^2)^2]/\Sigma[w(F_o^2)^2]]^{½}$. [c] Quality-of-fit = $[\Sigma[w(F_o^2-F_c^2)^2]/(N_{obs}-N_{params})]^{½}$, based on all data.

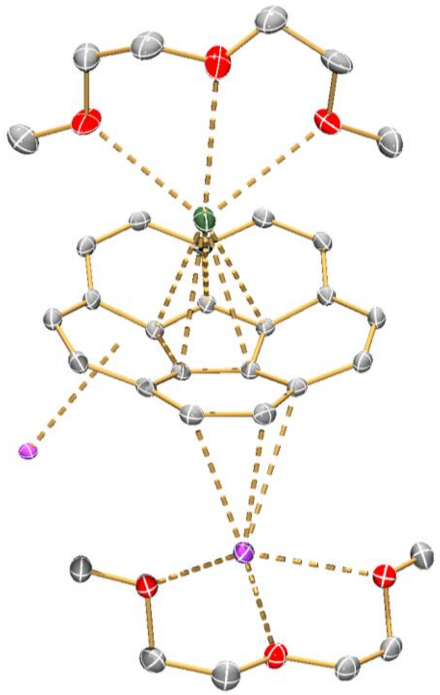

Figure S6. ORTEP drawing of the asymmetric unit of **1** with thermal ellipsoids shown at the 40% probability level. Color scheme used: cesium (green), potassium (purple), oxygen (red), carbon (gray). Hydrogen atoms are removed for clarity.

IV. Calculation details

Geometry optimization was performed at the DFT level of theory with help of parameter-free exchange-correlation functional PBE0. Light atoms (C, H, O) were described by the correlation-consistent basis sets of double-ζ quality (cc-pVDZ). Metal atoms were described by the triple-ζ basis sets of Stuttgart-Bonn group (def2-TZVP; in the case of Cs this basis set is equipped with effective small-core potential). All calculations were performed by using the Firefly program package (version 8.1.0).[9] The calculated structures correspond to the local minimum (no imaginary frequencies) on the corresponding potential energy surfaces, as determined by calculation of the full Hessian matrix followed by estimation of frequencies in the harmonic approximation. In the case of models **1H**-K-*small* and **1H**-K-*full*, only positions of hydrogen atoms were optimized, whereas positions of other atoms were taken from the crystal structure and kept frozen (Fig. S7). These calculations were performed with the ORCA program suite (version 3.0.3).[10] In this part, all atoms were described by relativistically re-contracted basis sets of triple-ζ quality (SARS-TZVP).[11] All calculations were carried out with RIJCOSX acceleration technique.[12] Scalar relativistic effects have been incorporated by applying the 0th-order regular approximation (ZORA). All optimized geometries were then used for subsequent analysis of the electronic structure of the product in terms of natural bond orbitals (NBO) approach.[13] All NBO computations were performed with the NBO 6.0 program.[14] Broken-symmetry (BS-PBE0) calculations were performed with help of ORCA package using Yamaguchi formula[15] for calculating J coupling constant. In all cases we used Heisenberg Hamiltonian in the form: $H = -2JS_1S_2$. Thus, the energy gap between singlet and triplet states was equal to $\Delta = 2J$.

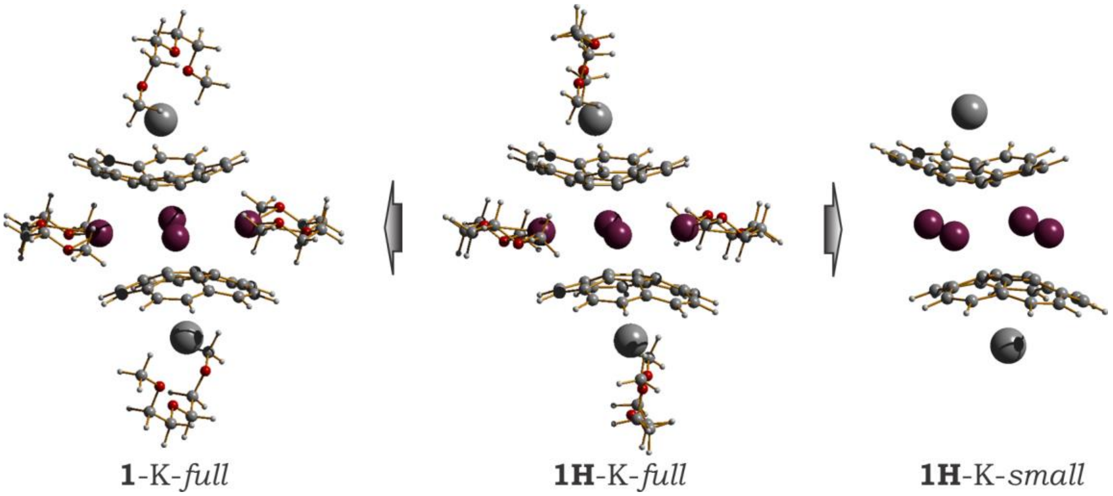

Figure S7. Equilibrium structures for models **1**-K-*small*, **1H**-K-*small*, and **1**-K-*full*.

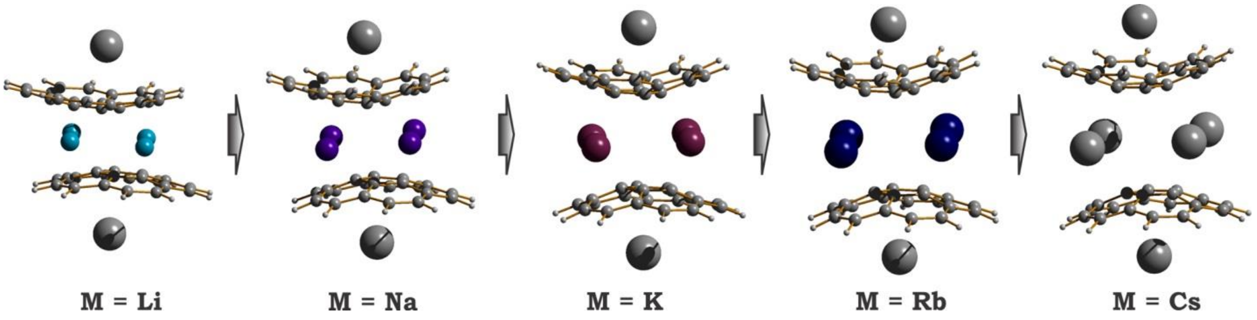

Figure S8. Equilibrium geometry configurations for **1**-M-*small* systems (M = Li–Cs).

Multireference calculations were performed at the level of multiconfigurational perturbation theory of the second in XMCQDPT2 variant.[16] The same basis sets were utilized as for the geometry optimization (def2-TZVP(*metal*)//cc-pVDZ(C,H,O)). The active space used for the reference CASSCF calculations was constructed by including all doubly and singly occupied orbitals on the top of neutral corannulene and 6 cesium cations plus adding four occupied MOs, which represent two degenerate HOMOs of the neutral corannulene. Totally those correspond to 14 shared over 8 orbitals or 14/8 approach (Figs. S9 and S10). An initial guess of the orbitals for CASSCF calculations was taken from the converged PBE0 calculations. CASSCF calculations

were performed using a state-average approach. The lowest-lying one singlet and one triplet states were considered (with ALDET keyword in Firefly terminology). The singlet state was corresponding to an open-shell singlet electronic state. The converged CASSCF wavefunction was further used as a reference wavefunction for the calculations by multireference Møller-Plesset perturbation theory of the second order (MRMP2). The MRMP2 model is a special state-specific case of the XMCQDPT2 theory that was recently developed by Granovsky.[16] The conventional intruder state avoidance (ISA) technique[17] was used in MRPT2 calculations.

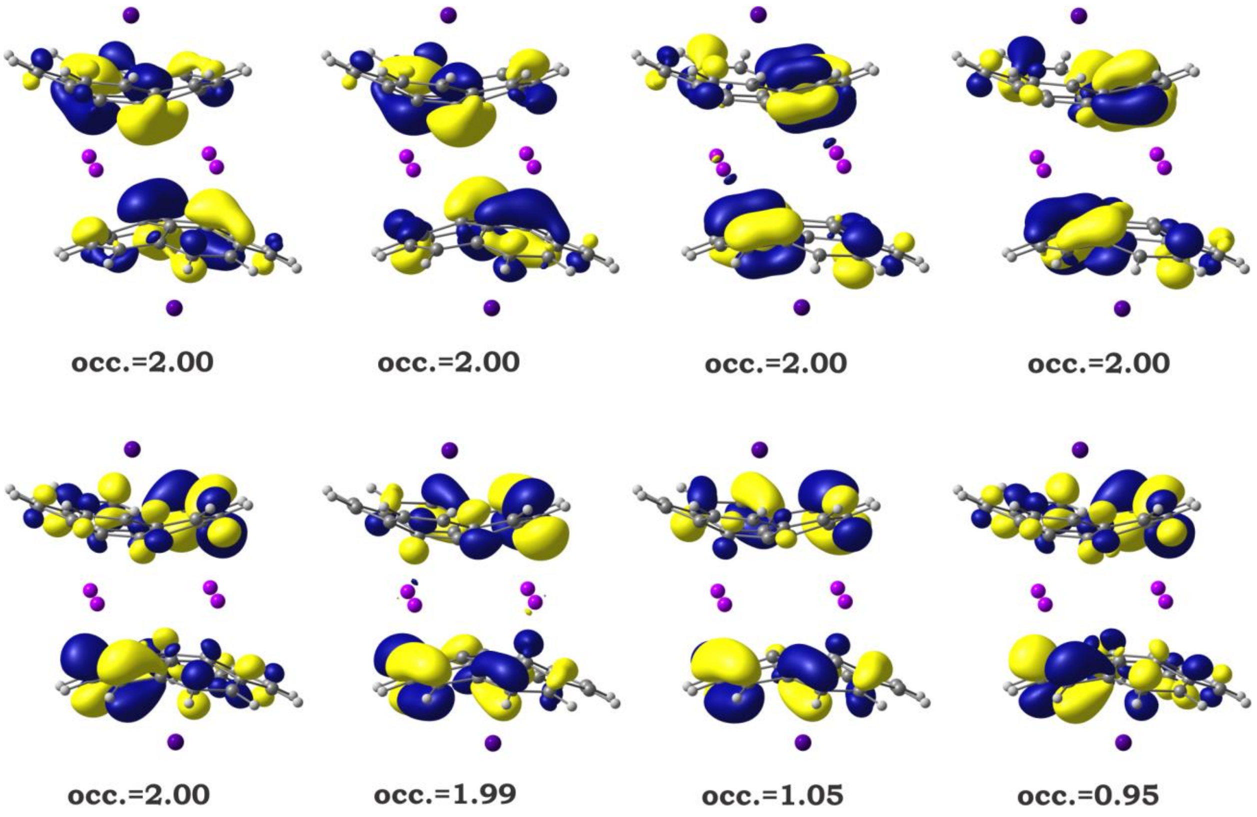

Figure S9. CASSCF(14,8) natural orbitals along with occupancies for **1H**-K-*small* model.

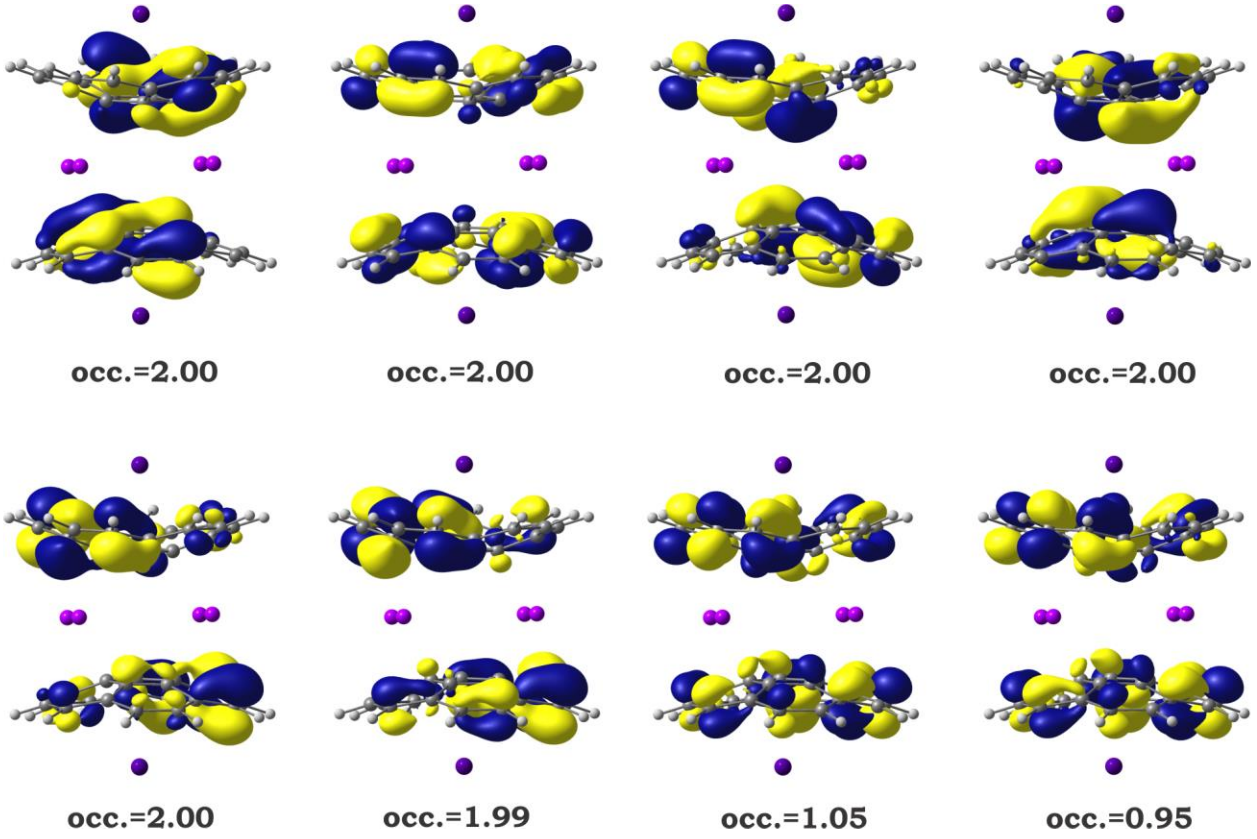

Figure S10. CASSCF(14,8) natural orbitals along with occupancies for **1**-K-*small* model.

Table S2. Cartesian coordinates for **1-K-***full* system, optimized at the PBE0/def2-TZVP(K,Cs)//cc-pVDZ(C,H,O) level of theory.

Cs	5.221427000	0.451361000	-0.008057000
K	-0.047679000	-0.432359000	-2.773263000
K	-0.466027000	3.420506000	-0.818797000
O	-0.370744000	4.462469000	-3.421097000
O	-1.405806000	6.001946000	-1.311832000
O	-0.299648000	5.350583000	1.179574000
C	2.689791000	1.155035000	-3.140803000
H	2.897658000	1.868854000	-3.946716000
C	2.927167000	-0.231580000	-3.426791000
H	3.282615000	-0.478933000	-4.432791000
C	2.911416000	-1.248840000	-2.405233000
C	3.465662000	-2.567174000	-2.308959000
H	3.863506000	-3.054900000	-3.205594000
C	3.680437000	-3.225618000	-1.051642000
H	4.255621000	-4.158851000	-1.070446000
C	3.307413000	-2.661576000	0.194058000
C	3.689206000	-2.913508000	1.563815000
H	4.234586000	-3.833278000	1.804870000
C	3.503543000	-1.980600000	2.608072000
H	3.931281000	-2.232533000	3.585380000
C	2.933616000	-0.658445000	2.409259000
C	3.015016000	0.554491000	3.132207000
H	3.438566000	0.561473000	4.142743000
C	2.718157000	1.850275000	2.528618000

H	2.944413000	2.739813000	3.126744000
C	2.374881000	1.993552000	1.151393000
C	2.472851000	3.093922000	0.215093000
H	2.685877000	4.100035000	0.592999000
C	2.483885000	2.919457000	-1.202829000
H	2.694840000	3.806570000	-1.812250000
C	2.351823000	1.642800000	-1.841251000
C	2.117647000	0.587984000	-0.896729000
C	2.112646000	0.753750000	0.498129000
C	2.382593000	-0.512235000	1.096633000
C	2.574039000	-1.441326000	0.064308000
C	2.412757000	-0.766511000	-1.166177000
C	-0.386000000	3.432414000	-4.387008000
H	-1.328453000	2.855194000	-4.338815000
H	0.464631000	2.772018000	-4.162201000
H	-0.263558000	3.843225000	-5.407327000
C	-1.407504000	5.397404000	-3.609681000
H	-2.392399000	4.909372000	-3.478923000
H	-1.362848000	5.824576000	-4.631561000
C	-1.244259000	6.515447000	-2.612510000
H	-0.239743000	6.969987000	-2.722336000
H	-1.999399000	7.299735000	-2.819897000
C	-1.206300000	6.963716000	-0.301647000
H	-1.959118000	7.773226000	-0.380237000
H	-0.201139000	7.418821000	-0.402361000
C	-1.331680000	6.298514000	1.044716000
H	-1.252068000	7.075030000	1.831929000
H	-2.319174000	5.807353000	1.139335000
C	-0.286777000	4.736983000	2.454093000
H	-0.116259000	5.487037000	3.250024000
H	0.546231000	4.018317000	2.454129000
H	-1.236727000	4.204601000	2.650031000
O	7.293367000	-0.450768000	-2.247011000
O	6.964622000	2.464349000	1.758166000
C	6.603985000	-0.950932000	-3.374098000
H	6.208020000	-0.129070000	-3.998753000
H	5.761157000	-1.552535000	-3.005722000
H	7.268692000	-1.583560000	-3.993318000
C	8.387238000	0.351062000	-2.605646000
H	8.051285000	1.244463000	-3.170521000
H	9.086411000	-0.213313000	-3.256012000
C	9.127851000	0.781240000	-1.367938000
H	9.391618000	-0.110373000	-0.764724000
H	10.072757000	1.277530000	-1.669161000
O	8.329581000	1.666038000	-0.617818000
C	8.981495000	2.112924000	0.546573000
H	9.942421000	2.602102000	0.287396000
H	9.207509000	1.259963000	1.217386000
C	8.111626000	3.107387000	1.267325000
H	8.696729000	3.550119000	2.099233000
H	7.833385000	3.929649000	0.577318000
C	6.144375000	3.333447000	2.507644000
H	6.671210000	3.696629000	3.410785000
H	5.249590000	2.768970000	2.802647000
H	5.830115000	4.206781000	1.905581000
K	0.047952000	0.432494000	2.773180000
K	0.465213000	-3.420405000	0.818852000
C	-3.465604000	2.567083000	2.308961000
C	-3.680624000	3.225568000	1.051651000
C	-3.307634000	2.661527000	-0.194010000
C	-2.933478000	0.658516000	-2.409362000
C	-2.718245000	-1.850233000	-2.528717000
C	-2.374716000	-1.993578000	-1.151454000
C	-2.112553000	-0.753720000	-0.498205000
C	-2.382692000	0.512214000	-1.096656000
C	-2.574231000	1.441257000	-0.064349000
C	-2.412765000	0.766401000	1.166150000
Cs	-5.221403000	-0.451290000	0.007953000
O	-8.329581000	-1.666056000	0.617830000
O	0.370737000	-4.462528000	3.421074000
O	1.405889000	-6.001992000	1.311883000

O	0.299723000	-5.350527000	-1.179626000
C	-2.689483000	-1.154923000	3.140927000
H	-2.897139000	-1.868649000	3.946975000
C	-2.926935000	0.231612000	3.426818000
H	-3.282193000	0.479136000	4.432811000
C	-2.911539000	1.248761000	2.405126000
H	-3.863508000	3.054709000	3.205600000
H	-4.255430000	4.158978000	1.070570000
C	-3.689175000	2.913628000	-1.563844000
H	-4.234429000	3.833447000	-1.804987000
C	-3.503754000	1.980563000	-2.607972000
H	-3.931318000	2.232537000	-3.585327000
C	-3.014835000	-0.554430000	-3.132320000
H	-3.438568000	-0.561368000	-4.142778000
H	-2.944563000	-2.739760000	-3.126902000
C	-2.473126000	-3.093855000	-0.215164000
H	-2.686371000	-4.099986000	-0.592910000
C	-2.484033000	-2.919369000	1.202849000
H	-2.695014000	-3.806567000	1.812142000
C	-2.351734000	-1.642794000	1.841248000
C	-2.117575000	-0.587986000	0.896726000
C	0.385902000	-3.432428000	4.387002000
H	1.328326000	-2.855143000	4.338809000
H	-0.464767000	-2.772098000	4.162194000
H	0.263505000	-3.843254000	5.407282000
C	1.407487000	-5.397401000	3.609746000
H	2.392442000	-4.909378000	3.479066000
H	1.362728000	-5.824541000	4.631609000
C	1.244240000	-6.515444000	2.612562000
H	0.239722000	-6.969933000	2.722455000
H	1.999375000	-7.299794000	2.820035000
C	1.206356000	-6.963717000	0.301619000
H	1.959098000	-7.773225000	0.380145000
H	0.201166000	-7.418800000	0.402250000
C	1.331777000	-6.298443000	-1.044721000
H	1.252136000	-7.075021000	-1.831965000
H	2.319229000	-5.807300000	-1.139367000
C	0.286873000	-4.736907000	-2.454128000
H	0.116381000	-5.486913000	-3.250095000
H	-0.546168000	-4.018272000	-2.454166000
H	1.236777000	-4.204438000	-2.650037000
O	-7.293485000	0.450780000	2.246967000
O	-6.964543000	-2.464428000	-1.758113000
C	-6.604086000	0.950967000	3.374025000
H	-6.208088000	0.129098000	3.998649000
H	-5.761274000	1.552579000	3.005636000
H	-7.268751000	1.583591000	3.993297000
C	-8.387341000	-0.351035000	2.605633000
H	-8.051352000	-1.244438000	3.170452000
H	-9.086538000	0.213315000	3.255996000
C	-9.127910000	-0.781277000	1.367895000
H	-9.391681000	0.110328000	0.764656000
H	-10.072818000	-1.277555000	1.669107000
C	-8.981444000	-2.113105000	-0.546520000
H	-9.942333000	-2.602393000	-0.287299000
H	-9.207575000	-1.260235000	-1.217380000
C	-8.111498000	-3.107534000	-1.267189000
H	-8.696542000	-3.550387000	-2.099046000
H	-7.833140000	-3.929704000	-0.577095000
C	-6.144271000	-3.333555000	-2.507584000
H	-6.671186000	-3.696789000	-3.410683000
H	-5.249552000	-2.769043000	-2.802662000
H	-5.829973000	-4.206826000	-1.905503000

Table S3. Cartesian coordinates for **1H-K-*full*** system, optimized at the PBE0/TZVP/ZORA level of theory.

Cs	5.181365000	0.601874000	-0.200976000
K	-0.107024000	-1.002074000	-3.054702000
K	-0.889220000	3.328273000	-0.312494000
O	-0.810534000	4.253608000	-2.927252000
O	-1.914508000	5.759634000	-0.841858000
O	-0.801754000	5.351223000	1.706002000
C	2.435650000	0.678810000	-2.888485000
H	2.600805000	1.212244000	-3.837711000
C	2.843924000	-0.716399000	-2.824019000
H	3.292830000	-1.144726000	-3.734739000
C	2.898896000	-1.449432000	-1.607863000
C	3.630093000	-2.631303000	-1.219146000
H	4.068910000	-3.272717000	-2.000328000
C	3.985425000	-2.919501000	0.132383000
H	4.660431000	-3.771793000	0.305383000
C	3.610005000	-2.081192000	1.235807000
C	4.177000000	-1.897607000	2.529109000
H	4.898351000	-2.636698000	2.912435000
C	3.966187000	-0.720659000	3.312577000
H	4.520197000	-0.635888000	4.259690000
C	3.198012000	0.403013000	2.840346000
C	3.152598000	1.782191000	3.223471000
H	3.630178000	2.106580000	4.160597000
C	2.674906000	2.819157000	2.330122000
H	2.843313000	3.862568000	2.640598000
C	2.179818000	2.549339000	1.032380000
C	2.049785000	3.360761000	-0.148418000
H	2.132290000	4.455988000	-0.057744000
C	1.981603000	2.822911000	-1.450681000
H	2.015224000	3.536643000	-2.287862000
C	2.047522000	1.404003000	-1.731419000
C	1.964331000	0.618576000	-0.546259000
C	2.054485000	1.153722000	0.745302000
C	2.532587000	0.139755000	1.610514000
C	2.712823000	-1.038596000	0.853543000
C	2.371135000	-0.735680000	-0.489163000
C	-0.848223000	3.094860000	-3.761152000
H	-1.761639000	2.489082000	-3.577518000
H	0.051315000	2.500050000	-3.523638000
H	-0.819506000	3.378638000	-4.836206000
C	-1.967842000	5.055634000	-3.106372000
H	-2.885235000	4.462124000	-2.893331000
H	-2.037232000	5.426231000	-4.155840000
C	-1.886523000	6.233980000	-2.176259000
H	-0.948528000	6.803780000	-2.366005000
H	-2.744197000	6.917889000	-2.372031000
C	-1.841210000	6.796430000	0.132094000
H	-2.674732000	7.523052000	-0.001640000
H	-0.883506000	7.355569000	0.034572000
C	-1.943124000	6.169077000	1.483294000
H	-2.007792000	6.969010000	2.257155000
H	-2.873083000	5.559866000	1.541661000
C	-0.831621000	4.705857000	2.975433000
H	-0.901932000	5.441303000	3.806123000
H	0.126789000	4.161697000	3.067527000
H	-1.685709000	3.996851000	3.050879000
O	7.108567000	-0.531925000	-2.438654000
O	7.351968000	1.478990000	1.847768000
C	6.390449000	-1.199149000	-3.368543000
H	5.936257000	-0.533407000	-4.139380000
H	5.566341000	-1.716335000	-2.848099000
H	6.994869000	-1.966783000	-3.911885000
C	8.277270000	0.208577000	-2.743483000
H	7.998289000	1.197427000	-3.175385000
H	8.846832000	-0.323677000	-3.543549000

C	9.210644000	0.444807000	-1.590322000
H	9.412099000	-0.505437000	-1.059368000
H	10.165074000	0.874410000	-1.957385000
O	8.518883000	1.400558000	-0.701828000
C	9.302281000	1.190181000	0.514879000
H	10.359061000	1.495018000	0.360536000
H	9.263783000	0.129484000	0.831622000
C	8.635391000	2.052231000	1.592347000
H	9.253461000	2.038544000	2.517619000
H	8.545041000	3.106441000	1.251860000
C	6.840134000	1.900240000	3.073073000
H	7.487222000	1.582491000	3.921572000
H	5.841738000	1.448371000	3.200629000
H	6.725341000	3.005633000	3.123145000
K	0.107035000	1.002060000	3.054692000
K	0.889230000	-3.328286000	0.312483000
C	-3.630083000	2.631290000	1.219135000
C	-3.985415000	2.919488000	-0.132394000
C	-3.609994000	2.081178000	-1.235818000
C	-3.198001000	-0.403027000	-2.840356000
C	-2.674895000	-2.819171000	-2.330132000
C	-2.179808000	-2.549353000	-1.032391000
C	-2.054474000	-1.153736000	-0.745312000
C	-2.532576000	-0.139769000	-1.610526000
C	-2.712812000	1.038583000	-0.853554000
C	-2.371124000	0.735666000	0.489153000
Cs	-5.181354000	-0.601888000	0.200965000
O	-8.518873000	-1.400572000	0.701816000
O	0.810545000	-4.253622000	2.927241000
O	1.914519000	-5.759648000	0.841847000
O	0.801764000	-5.351237000	-1.706014000
C	-2.435640000	-0.678824000	2.888473000
H	-2.600588000	-1.212233000	3.837753000
C	-2.843914000	0.716386000	2.824008000
H	-3.293530000	1.144534000	3.734474000
C	-2.898885000	1.449418000	1.607853000
H	-4.068056000	3.273063000	2.000513000
H	-4.660180000	3.771961000	-0.305452000
C	-4.176990000	1.897593000	-2.529120000
H	-4.898807000	2.636424000	-2.912077000
C	-3.966176000	0.720645000	-3.312588000
H	-4.520103000	0.636012000	-4.259760000
C	-3.152587000	-1.782205000	-3.223483000
H	-3.630101000	-2.106542000	-4.160662000
H	-2.843313000	-3.862590000	-2.640571000
C	-2.049774000	-3.360774000	0.148408000
H	-2.132268000	-4.456005000	0.057783000
C	-1.981593000	-2.822924000	1.450670000
H	-2.015174000	-3.536641000	2.287867000
C	-2.047510000	-1.404017000	1.731409000
C	-1.964320000	-0.618590000	0.546249000
C	0.848234000	-3.094874000	3.761142000
H	1.761698000	-2.489144000	3.577578000
H	-0.051247000	-2.500011000	3.523549000
H	0.819400000	-3.378647000	4.836193000
C	1.967853000	-5.055648000	3.106362000
H	2.885244000	-4.462140000	2.893313000
H	2.037244000	-5.426244000	4.155829000
C	1.886534000	-6.233995000	2.176248000
H	0.948541000	-6.803800000	2.365994000
H	2.744208000	-6.917902000	2.372022000
C	1.841220000	-6.796443000	-0.132104000
H	2.674744000	-7.523064000	0.001629000
H	0.883516000	-7.355584000	-0.034580000
C	1.943135000	-6.169091000	-1.483305000
H	2.007801000	-6.969024000	-2.257166000
H	2.873088000	-5.559869000	-1.541675000
C	0.831631000	-4.705871000	-2.975444000
H	0.901937000	-5.441313000	-3.806137000
H	-0.126774000	-4.161699000	-3.067539000
H	1.685738000	-3.996886000	-3.050866000

O	-7.108556000	0.531911000	2.438643000
O	-7.351957000	-1.479004000	-1.847779000
C	-6.390438000	1.199134000	3.368533000
H	-5.908524000	0.530678000	4.119894000
H	-5.586741000	1.744147000	2.844444000
H	-7.003787000	1.942451000	3.935175000
C	-8.277258000	-0.208591000	2.743473000
H	-7.998731000	-1.197667000	3.175231000
H	-8.846213000	0.324028000	3.543747000
C	-9.210634000	-0.444820000	1.590311000
H	-9.412042000	0.505409000	1.059305000
H	-10.165034000	-0.874409000	1.957461000
C	-9.302270000	-1.190195000	-0.514890000
H	-10.359036000	-1.495083000	-0.360555000
H	-9.263808000	-0.129493000	-0.831609000
C	-8.635380000	-2.052245000	-1.592357000
H	-9.253467000	-2.038576000	-2.517617000
H	-8.544995000	-3.106433000	-1.251817000
C	-6.840124000	-1.900253000	-3.073083000
H	-7.485977000	-1.580497000	-3.921756000
H	-5.840705000	-1.450346000	-3.199638000
H	-6.727345000	-3.005819000	-3.123792000

Table S4. Cartesian coordinates for **1H-K-*small*** system, optimized at the PBE0/TZVP/ZORA level of theory.

Cs	-5.179945000	-0.027902000	-0.646302000
K	0.342605000	2.486882000	-2.011202000
K	0.760831000	-2.680992000	-2.049256000
C	-2.275947000	1.169149000	-2.860938000
H	-2.387545000	1.253864000	-3.953061000
C	-2.627547000	2.331798000	-2.059041000
H	-3.000766000	3.217591000	-2.598004000
C	-2.725420000	2.290763000	-0.642050000
C	-3.427857000	3.121754000	0.306922000
H	-3.791767000	4.109591000	-0.015149000
C	-3.854555000	2.655864000	1.586542000
H	-4.508022000	3.321088000	2.172864000
C	-3.586661000	1.329264000	2.065202000
C	-4.241283000	0.514793000	3.031981000
H	-4.939023000	0.978898000	3.746081000
C	-4.131112000	-0.910008000	3.054432000
H	-4.752145000	-1.455210000	3.780368000
C	-3.385240000	-1.650117000	2.068544000
C	-3.424231000	-3.015764000	1.639475000
H	-3.990421000	-3.756215000	2.223514000
C	-2.937028000	-3.434038000	0.340104000
H	-3.164397000	-4.467226000	0.033905000
C	-2.349445000	-2.539915000	-0.587241000
C	-2.181054000	-2.588537000	-2.014470000
H	-2.374580000	-3.539901000	-2.539841000
C	-2.007869000	-1.437355000	-2.810991000
H	-2.065750000	-1.567069000	-3.904252000
C	-1.994119000	-0.092313000	-2.275809000
C	-1.950929000	-0.082580000	-0.851308000
C	-2.146571000	-1.224394000	-0.063344000
C	-2.632441000	-0.810363000	1.199997000
C	-2.712926000	0.599196000	1.202798000
C	-2.302186000	1.049533000	-0.078030000
K	-0.342605000	-2.486883000	2.011202000
K	-0.760832000	2.680992000	2.049256000
C	3.427857000	-3.121753000	-0.306922000
C	3.854556000	-2.655864000	-1.586543000
C	3.586661000	-1.329264000	-2.065203000
C	3.385240000	1.650117000	-2.068544000
C	2.937028000	3.434038000	-0.340103000

C	2.349446000	2.539916000	0.587241000
C	2.146572000	1.224395000	0.063343000
C	2.632442000	0.810363000	-1.199997000
C	2.712926000	-0.599197000	-1.202798000
C	2.302186000	-1.049533000	0.078030000
Cs	5.179944000	0.027902000	0.646303000
C	2.275947000	-1.169148000	2.860938000
H	2.387545000	-1.253863000	3.953061000
C	2.627547000	-2.331797000	2.059040000
H	3.000766000	-3.217591000	2.598003000
C	2.725420000	-2.290762000	0.642050000
H	3.791767000	-4.109590000	0.015149000
H	4.508022000	-3.321088000	-2.172865000
C	4.241283000	-0.514793000	-3.031981000
H	4.939022000	-0.978898000	-3.746081000
C	4.131113000	0.910008000	-3.054432000
H	4.752146000	1.455210000	-3.780369000
C	3.424232000	3.015764000	-1.639475000
H	3.990421000	3.756215000	-2.223514000
H	3.164398000	4.467226000	-0.033905000
C	2.181055000	2.588537000	2.014469000
H	2.374581000	3.539901000	2.539840000
C	2.007869000	1.437355000	2.810992000
H	2.065749000	1.567069000	3.904252000
C	1.994120000	0.092313000	2.275809000
C	1.950930000	0.082580000	0.851307000

Table S5. Cartesian coordinates for **1**-Li-*small* system, optimized at the PBE0/def2-TZVP(Li,Cs)//cc-pVDZ(C,H,O) level of theory.

Cs	4.445976437	-0.070248698	-0.695790851
Li	-0.392777499	-2.460619124	-1.415002630
C	2.213590144	3.403597203	0.143433547
H	2.350984242	4.431619706	-0.201827583
C	2.554410930	3.137090617	1.533106612
H	2.917586206	3.986958502	2.117951235
C	2.506735566	1.837729689	2.103206117
C	2.989073700	1.189397659	3.315437135
H	3.401315823	1.805291023	4.120563464
C	3.011997785	-0.198007034	3.501222485
H	3.440461528	-0.567319578	4.437950061
C	2.554348785	-1.158223351	2.505745825
C	2.646895737	-2.559734126	2.298672277
H	3.038070682	-3.213047727	3.083807655
C	2.318511282	-3.193524656	1.029518448
H	2.492539362	-4.270690186	0.965322363
C	1.911482849	-2.464651719	-0.138316289
C	1.792145633	-2.725159052	-1.559490026
H	2.000856527	-3.726754672	-1.948491829
C	1.612773885	-1.666337769	-2.543844795
H	1.658643234	-1.968721579	-3.595073332
C	1.473154180	-0.282295693	-2.204532308
C	1.570205641	0.967179807	-2.897404570
H	1.610359623	0.982272321	-3.991412721
C	1.712034978	2.252803757	-2.228537430
H	1.897074898	3.121163096	-2.868820602
C	1.835942739	2.380154686	-0.789210370
C	1.636635641	1.133977939	-0.148403596
C	1.976226799	0.883406753	1.200024609
C	1.995336925	-0.493417092	1.385714357
C	1.667706824	-1.100714519	0.152365613
C	1.432579224	-0.092852126	-0.790299069
Li	0.469914975	-2.013161938	2.026457210
Cs	-4.445871999	0.070419296	0.696211954
Li	0.392690391	2.460392066	1.415616839
C	-2.213377670	-3.403595080	-0.142842547

H	-2.350861545	-4.431571842	0.202587693
C	-2.554207403	-3.137278148	-1.532606173
H	-2.917199303	-3.987249935	-2.117303170
C	-2.506950088	-1.837906626	-2.102791655
C	-2.989229642	-1.189773528	-3.315225298
H	-3.401440140	-1.805929781	-4.120185653
C	-3.012119596	0.197558483	-3.501370460
H	-3.440697653	0.566600711	-4.438180879
C	-2.554406690	1.157922682	-2.506076415
C	-2.647046585	2.559382714	-2.299060913
H	-3.038029715	3.212644159	-3.084314659
C	-2.318585122	3.193351768	-1.030087730
H	-2.492569740	4.270559465	-0.966080984
C	-1.911733228	2.464705345	0.137961449
C	-1.792389910	2.725521065	1.558985874
H	-2.001239927	3.727108373	1.947956063
C	-1.612623223	1.666828173	2.543511976
H	-1.658805168	1.969307831	3.594691521
C	-1.473081905	0.282797943	2.204370229
C	-1.570102536	-0.966660400	2.897437016
H	-1.609896965	-0.981496191	3.991398221
C	-1.711824946	-2.252402895	2.228914202
H	-1.896332913	-3.120844618	2.869270938
C	-1.835801579	-2.380024995	0.789566121
C	-1.636760633	-1.133857763	0.148619608
C	-1.976512237	-0.883454332	-1.199799390
C	-1.995481619	0.493314903	-1.385850069
C	-1.667826788	1.100795627	-0.152621027
C	-1.432638939	0.093063273	0.790199614
Li	-0.469840269	2.012610137	-2.026943305

Table S6. Cartesian coordinates for **1**-Na-*small* system, optimized at the PBE0/def2-TZVP(Na,Cs)//cc-pVDZ(C,H,O) level of theory.

Cs	4.731971395	0.001437502	-0.537080055
Na	-0.152977491	-2.374676366	-1.522875101
C	2.579248332	3.360554964	-0.016622567
H	2.799591725	4.370625737	-0.376303648
C	2.819718880	3.101499962	1.356797801
H	3.205633371	3.931561069	1.957793257
C	2.701265452	1.785325665	1.950076150
C	3.195982352	1.167777858	3.128562493
H	3.654769393	1.782353310	3.909561957
C	3.223284556	-0.250550664	3.316807475
H	3.701017644	-0.624995679	4.226954986
C	2.764289659	-1.175264956	2.331186793
C	2.919114398	-2.601654306	2.118572430
H	3.345042215	-3.221903285	2.912926570
C	2.724130613	-3.217094142	0.848826130
H	3.006522606	-4.271769083	0.762654812
C	2.282152305	-2.511080732	-0.318579547
C	2.377969480	-2.743210574	-1.724348135
H	2.685941317	-3.727801468	-2.093686138
C	2.183159232	-1.710869870	-2.710680928
H	2.382370425	-1.980197465	-3.752177834
C	1.968136091	-0.316540914	-2.367924333
C	2.169388251	0.912255415	-3.063576136
H	2.400545444	0.897078066	-4.132918554
C	2.297761142	2.204069244	-2.380206423
H	2.596255543	3.060874961	-2.992979318
C	2.198188226	2.335029379	-0.974651956
C	1.846449098	1.111482850	-0.316314296
C	2.123605998	0.855679590	1.034679958
C	2.150966408	-0.541188529	1.218946223
C	1.878787725	-1.162397328	-0.016732720
C	1.713629445	-0.141778000	-0.975124202

Na	0.202841365	-1.770033189	2.256086126
Cs	-4.731989016	-0.001187282	0.537690279
Na	0.153117656	2.375284454	1.522194339
C	-2.579021810	-3.360683238	0.016317015
H	-2.799197116	-4.370745812	0.375974825
C	-2.819759157	-3.101468991	-1.357007884
H	-3.205482260	-3.931597269	-1.958030727
C	-2.700928260	-1.785597118	-1.950291810
C	-3.195430529	-1.167882939	-3.128924924
H	-3.653932897	-1.782441369	-3.910097983
C	-3.222960640	0.250217922	-3.316954315
H	-3.700309980	0.624751196	-4.227254483
C	-2.764224057	1.175212432	-2.331024623
C	-2.919092533	2.601381026	-2.118581530
H	-3.345414476	3.221618957	-2.912859322
C	-2.724354466	3.216938692	-0.848499965
H	-3.007298933	4.271442051	-0.762216200
C	-2.282238119	2.510999953	0.318493059
C	-2.377787195	2.743132262	1.724630939
H	-2.685552424	3.727831082	2.093951491
C	-2.183152357	1.710968896	2.710751742
H	-2.382049898	1.980400078	3.752326498
C	-1.968249742	0.316361671	2.368161219
C	-2.170207279	-0.912085883	3.063553646
H	-2.401676117	-0.896859721	4.132815566
C	-2.297721906	-2.204208060	2.380188890
H	-2.596342600	-3.061045987	2.992904726
C	-2.198352697	-2.334943648	0.974557143
C	-1.846818580	-1.111516213	0.316275220
C	-2.123696129	-0.855757337	-1.034746448
C	-2.151006101	0.540971715	-1.218906097
C	-1.878763317	1.162312765	0.016892057
C	-1.713621405	0.141799816	0.975197648
Na	-0.203238258	1.771796873	-2.255131264

Table S7. Cartesian coordinates for **1**-K-*small* system, optimized at the PBE0/def2-TZVP(K,Cs)//cc-pVDZ(C,H,O) level of theory.

Cs	5.033072933	0.036400869	-0.502306730
K	-0.115793056	-2.605402386	-1.626573214
C	2.921141273	3.336007401	-0.117366746
H	3.207812848	4.328601313	-0.480131160
C	3.120178575	3.070625842	1.260635747
H	3.531438969	3.883176454	1.870375330
C	2.921628177	1.770023603	1.851527603
C	3.367220267	1.153922315	3.050742959
H	3.812839579	1.768748157	3.840040290
C	3.385442100	-0.262968792	3.240823834
H	3.847516579	-0.639739357	4.158682148
C	2.988133106	-1.186291093	2.221372832
C	3.205515302	-2.596280554	2.011015425
H	3.647984464	-3.199734260	2.810044345
C	3.061838825	-3.211717204	0.731299800
H	3.401080417	-4.249449116	0.638571666
C	2.630942413	-2.511548216	-0.434943004
C	2.814313960	-2.734405519	-1.839073839
H	3.172016418	-3.710246348	-2.188485834
C	2.652788528	-1.712899404	-2.823754208
H	2.923579131	-1.967724623	-3.853464626
C	2.385589500	-0.322871162	-2.490251423
C	2.656245557	0.896344055	-3.167002010
H	2.974897923	0.872770634	-4.214286253
C	2.746228022	2.182149511	-2.477461682
H	3.108781635	3.035727198	-3.060231924
C	2.549201444	2.313211723	-1.082754661
C	2.132235471	1.101073455	-0.441664793

C	2.343502797	0.844796120	0.923749151
C	2.380963970	-0.554868138	1.102613920
C	2.173218036	-1.176238341	-0.149153872
C	2.038940770	-0.155320192	-1.112002045
K	0.149167622	-1.766721606	2.546817570
Cs	-5.033068181	-0.036532138	0.502394777
K	0.115800088	2.605444121	1.626621007
C	-2.921174244	-3.336041235	0.117346097
H	-3.207854597	-4.328607306	0.480066483
C	-3.120154771	-3.070653856	-1.260628304
H	-3.531334465	-3.883176332	-1.870425679
C	-2.921625580	-1.769991279	-1.851519371
C	-3.367184285	-1.153904873	-3.050756648
H	-3.812766050	-1.768762840	-3.840049544
C	-3.385421955	0.262981136	-3.240840173
H	-3.847521636	0.639761316	-4.158712724
C	-2.988160582	1.186289982	-2.221352408
C	-3.205600881	2.596285748	-2.010992089
H	-3.648021428	3.199770173	-2.810004013
C	-3.061847359	3.211718576	-0.731252485
H	-3.401065639	4.249476856	-0.638566231
C	-2.630941432	2.511569979	0.434966820
C	-2.814323221	2.734406430	1.839082838
H	-3.172040745	3.710256467	2.188513290
C	-2.652742495	1.712908970	2.823761256
H	-2.923581736	1.967700242	3.853439720
C	-2.385577546	0.322839902	2.490239864
C	-2.656253071	-0.896349025	3.166988009
H	-2.974882718	-0.872769195	4.214233939
C	-2.746258413	-2.182151468	2.477443354
H	-3.108809485	-3.035740279	3.060195947
C	-2.549195270	-2.313242352	1.082740690
C	-2.132210554	-1.101082251	0.441671551
C	-2.343497814	-0.844764613	-0.923750701
C	-2.380982218	0.554874349	-1.102610002
C	-2.173237533	1.176247916	0.149141667
C	-2.038946960	0.155302665	1.111982252
K	-0.149180781	1.766781877	-2.546773786

Table S8. Cartesian coordinates for **1**-Rb-*small* system, optimized at the PBE0/def2-TZVP(Rb,Cs)//cc-pVDZ(C,H,O) level of theory.

Cs	5.173961280	0.033924735	-0.436799410
Rb	-0.113177849	-2.707567986	-1.716239662
C	3.085114383	3.330852991	-0.140156924
H	3.394891316	4.315747670	-0.505457935
C	3.278243033	3.062262677	1.237779269
H	3.705728247	3.866397949	1.848354375
C	3.052221986	1.769347342	1.828862387
C	3.497154233	1.153806869	3.028971442
H	3.956507828	1.767655038	3.811769288
C	3.508285272	-0.262014221	3.221881144
H	3.980125639	-0.640065844	4.134563722
C	3.112495103	-1.184986343	2.198592557
C	3.355512272	-2.587302174	1.987679452
H	3.816793938	-3.181203542	2.783754776
C	3.222136190	-3.203833065	0.704929550
H	3.589008193	-4.231770165	0.606795476
C	2.785412226	-2.506187649	-0.457157868
C	2.990404409	-2.727747759	-1.860604074
H	3.360974532	-3.701764538	-2.202550375
C	2.840679597	-1.709556200	-2.845033878
H	3.132967955	-1.960010943	-3.870369063
C	2.558937773	-0.321741247	-2.513832377
C	2.846695267	0.893758644	-3.185727972
H	3.189192646	0.866473091	-4.225704237

C	2.928853446	2.176899028	-2.495735858
H	3.311097932	3.028597251	-3.069058355
C	2.709747890	2.308240384	-1.104126831
C	2.273041958	1.101395138	-0.467424454
C	2.458887717	0.846578407	0.903755894
C	2.495132145	-0.554837911	1.082717670
C	2.309339597	-1.176374813	-0.175346190
C	2.189502162	-0.155350913	-1.140032879
Rb	0.143698533	-1.823036859	2.669138111
Cs	-5.173962139	-0.033909339	0.436790222
Rb	0.113176637	2.707577877	1.716219483
C	-3.085114508	-3.330846027	0.140153961
H	-3.394874139	-4.315737472	0.505429175
C	-3.278252432	-3.062278282	-1.237810937
H	-3.705702313	-3.866406247	-1.848380294
C	-3.052227387	-1.769324396	-1.828865465
C	-3.497158013	-1.153805183	-3.028961045
H	-3.956557844	-1.767641690	-3.811752586
C	-3.508301321	0.262023507	-3.221850871
H	-3.980179531	0.640088314	-4.134526779
C	-3.112504062	1.185009657	-2.198590674
C	-3.355492877	2.587326831	-1.987677062
H	-3.816744573	3.181247600	-2.783765843
C	-3.222127901	3.203848965	-0.704944027
H	-3.588966964	4.231795803	-0.606788957
C	-2.785405923	2.506179613	0.457199381
C	-2.990419731	2.727739414	1.860597902
H	-3.361004371	3.701732774	2.202557607
C	-2.840678332	1.709519211	2.845062503
H	-3.132992253	1.959986372	3.870375316
C	-2.558951312	0.321742480	2.513831286
C	-2.846658668	-0.893776962	3.185749855
H	-3.189167267	-0.866478525	4.225713292
C	-2.928884067	-2.176893310	2.495736447
H	-3.311090613	-3.028579714	3.069049299
C	-2.709733979	-2.308257658	1.104113286
C	-2.273016707	-1.101393728	0.467418136
C	-2.458905861	-0.846580172	-0.903752815
C	-2.495122342	0.554850042	-1.082678777
C	-2.309323001	1.176377388	0.175327150
C	-2.189531707	0.155338106	1.140042923
Rb	-0.143691347	1.822939714	-2.669207863

Table S9. Cartesian coordinates for **1-**Cs-*small* system, optimized at the PBE0/def2-TZVP(,Cs)//cc-pVDZ(C,H,O) level of theory.

Cs	5.293312237	0.041387413	-0.354440047
Cs	-0.117419400	-2.762787424	-1.883212716
C	3.223404158	3.335688315	-0.198425198
H	3.562490766	4.310830341	-0.562730510
C	3.400349548	3.060869813	1.184146787
H	3.836969893	3.856323905	1.800219549
C	3.132829338	1.785066809	1.770845915
C	3.541138298	1.166011647	2.990473408
H	3.983396344	1.780470849	3.783182481
C	3.529761249	-0.239801482	3.189910000
H	3.973003318	-0.620255774	4.116050685
C	3.161312349	-1.169434765	2.149382236
C	3.429794984	-2.556000972	1.944137564
H	3.890322829	-3.142757625	2.746284070
C	3.302704531	-3.185531942	0.658327757
H	3.685391871	-4.207703745	0.564024987
C	2.891437192	-2.484779811	-0.505928392
C	3.122508580	-2.712126323	-1.912033926
H	3.490026221	-3.690950103	-2.242645192
C	3.008986572	-1.696940406	-2.896657423

H	3.315168088	-1.953100493	-3.916916651
C	2.710665418	-0.312085836	-2.579533367
C	3.022422486	0.898879744	-3.243613745
H	3.371003481	0.871283083	-4.281811189
C	3.104393846	2.174870836	-2.553860170
H	3.496757080	3.025651216	-3.121535833
C	2.864399740	2.309600952	-1.160862202
C	2.398701912	1.113307263	-0.536023918
C	2.530793477	0.863656794	0.846764541
C	2.555756561	-0.535207762	1.028471413
C	2.415900584	-1.157222045	-0.240454758
C	2.325099467	-0.140977115	-1.209160725
Cs	0.114231952	-1.970148506	2.711431155
Cs	-5.293326814	-0.041380625	0.354227002
Cs	0.117448903	2.762492175	1.883772700
C	-3.223492321	-3.335604247	0.198463727
H	-3.562723307	-4.310643337	0.562751499
C	-3.400379642	-3.060861676	-1.184162613
H	-3.836998723	-3.856226091	-1.800319617
C	-3.132867458	-1.785039589	-1.770814614
C	-3.541098971	-1.165995742	-2.990566456
H	-3.983347406	-1.780379942	-3.783274129
C	-3.529614877	0.239755747	-3.190071347
H	-3.972932892	0.620244590	-4.116221536
C	-3.161315621	1.169412957	-2.149428191
C	-3.429940334	2.555960283	-1.944168920
H	-3.890292732	3.142756373	-2.746428354
C	-3.302658530	3.185500989	-0.658299108
H	-3.685637301	4.207572794	-0.563907123
C	-2.891501786	2.484694478	0.505942036
C	-3.122472695	2.712100681	1.912091290
H	-3.490187328	3.690929949	2.242530103
C	-3.009012020	1.696874092	2.896689508
H	-3.315103807	1.953197243	3.916888306
C	-2.710560296	0.312017996	2.579626925
C	-3.022433413	-0.898909292	3.243577755
H	-3.370854483	-0.871364817	4.281853236
C	-3.104236711	-2.174805808	2.553886499
H	-3.496712699	-3.025551254	3.121507778
C	-2.864427032	-2.309557333	1.160798087
C	-2.398721865	-1.113281900	0.535994329
C	-2.530724296	-0.863661484	-0.846792295
C	-2.555614622	0.535200960	-1.028533216
C	-2.415994975	1.157176438	0.240478049
C	-2.325051613	0.140911778	1.209149478
Cs	-0.114227306	1.970376763	-2.711047375

Table S10. NBO charges in **1**-K-*full* system, optimized at the PBE0/def2-TZVP(K,Cs)//cc-pVDZ(C,H,O) level of theory.

Atom	No	Natural Charge	Natural Population				Natural Spin Density
			Core	Valence	Rydberg	Total	
Cs	1	0.92294	53.98872	0.04428	0.04406	54.07706	-0.00047
K	2	0.94552	17.98943	0.03048	0.03457	18.05448	0.00479
K	3	0.89222	17.98965	0.06316	0.05496	18.10778	0.00165
O	4	-0.62584	1.99979	6.61511	0.01094	8.62584	0.00015
O	5	-0.62748	1.99977	6.61601	0.01170	8.62748	0.00009
O	6	-0.62803	1.99979	6.61736	0.01088	8.62803	0.00011
C	7	-0.32672	1.99900	4.30681	0.02091	6.32672	0.18025
H	8	0.22414	0.00000	0.77258	0.00328	0.77586	-0.00551
C	9	-0.40817	1.99902	4.38637	0.02278	6.40817	-0.01941
H	10	0.22456	0.00000	0.77233	0.00310	0.77544	0.00054
C	11	-0.11254	1.99876	4.09355	0.02023	6.11254	0.10665
C	12	-0.38414	1.99900	4.36373	0.02140	6.38414	-0.04255
H	13	0.22156	0.00000	0.77530	0.00314	0.77844	0.00119

C 14	-0.30017	1.99897	4.28154	0.01966	6.30017	0.08822
H 15	0.21965	0.00000	0.77704	0.00330	0.78035	-0.00279
C 16	-0.17724	1.99876	4.15694	0.02153	6.17724	-0.00523
C 17	-0.30175	1.99899	4.28268	0.02009	6.30175	0.12963
H 18	0.22328	0.00000	0.77342	0.00330	0.77672	-0.00398
C 19	-0.33009	1.99899	4.31057	0.02053	6.33009	0.08822
H 20	0.22249	0.00000	0.77418	0.00333	0.77751	-0.00261
C 21	-0.14865	1.99877	4.12844	0.02145	6.14865	0.04323
C 22	-0.39082	1.99899	4.36942	0.02240	6.39082	0.03045
H 23	0.22660	0.00000	0.77036	0.00304	0.77340	-0.00102
C 24	-0.44483	1.99902	4.42166	0.02415	6.44483	-0.00147
H 25	0.22272	0.00000	0.77417	0.00311	0.77728	-0.00002
C 26	-0.12094	1.99876	4.10176	0.02042	6.12094	0.07473
C 27	-0.39224	1.99901	4.37045	0.02278	6.39224	0.04696
H 28	0.22717	0.00000	0.76952	0.00331	0.77283	-0.00141
C 29	-0.33074	1.99900	4.31042	0.02132	6.33074	0.19185
H 30	0.22818	0.00000	0.76860	0.00323	0.77182	-0.00585
C 31	-0.17981	1.99877	4.15966	0.02138	6.17981	-0.05891
C 32	-0.16002	1.99873	4.14102	0.02027	6.16002	-0.00344
C 33	-0.13010	1.99874	4.11111	0.02025	6.13010	0.05750
C 34	-0.16531	1.99874	4.14624	0.02033	6.16531	0.02770
C 35	-0.14994	1.99874	4.13035	0.02085	6.14994	0.00618
C 36	-0.12708	1.99876	4.10892	0.01941	6.12708	0.06918
C 37	-0.27570	1.99936	4.26038	0.01597	6.27570	0.00162
H 38	0.20032	0.00000	0.79547	0.00421	0.79968	0.00072
H 39	0.21797	0.00000	0.77794	0.00409	0.78203	0.00035
H 40	0.18160	0.00000	0.81472	0.00368	0.81840	0.00011
C 41	-0.08900	1.99917	4.07056	0.01927	6.08900	0.00018
H 42	0.21314	0.00000	0.78085	0.00601	0.78686	-0.00004
H 43	0.19787	0.00000	0.79772	0.00440	0.80213	0.00001
C 44	-0.08334	1.99917	4.06493	0.01924	6.08334	0.00005
H 45	0.19901	0.00000	0.79524	0.00575	0.80099	-0.00001
H 46	0.19902	0.00000	0.79667	0.00431	0.80098	0.00001
C 47	-0.08337	1.99917	4.06501	0.01919	6.08337	0.00011
H 48	0.19861	0.00000	0.79711	0.00427	0.80139	0.00001
H 49	0.19843	0.00000	0.79580	0.00577	0.80157	-0.00001
C 50	-0.08892	1.99916	4.07038	0.01938	6.08892	0.00046
H 51	0.19697	0.00000	0.79858	0.00445	0.80303	0.00000
H 52	0.21497	0.00000	0.77913	0.00590	0.78503	0.00013
C 53	-0.27858	1.99934	4.26325	0.01598	6.27858	0.00108
H 54	0.17892	0.00000	0.81743	0.00365	0.82108	0.00013
H 55	0.21814	0.00000	0.77758	0.00428	0.78186	0.00034
H 56	0.20840	0.00000	0.78748	0.00411	0.79160	0.00005
O 57	-0.61955	1.99978	6.60891	0.01085	8.61955	0.00001
O 58	-0.61741	1.99978	6.60675	0.01088	8.61741	0.00001
C 59	-0.27352	1.99934	4.25876	0.01542	6.27352	-0.00014
H 60	0.18948	0.00000	0.80674	0.00378	0.81052	-0.00001
H 61	0.23463	0.00000	0.76205	0.00332	0.76537	0.00002
H 62	0.17773	0.00000	0.81846	0.00381	0.82227	0.00001
C 63	-0.08656	1.99918	4.06806	0.01932	6.08656	0.00000
H 64	0.19932	0.00000	0.79451	0.00616	0.80068	0.00000
H 65	0.19802	0.00000	0.79716	0.00482	0.80198	0.00000
C 66	-0.08882	1.99917	4.07009	0.01955	6.08882	0.00000
H 67	0.19962	0.00000	0.79433	0.00606	0.80038	0.00000
H 68	0.19716	0.00000	0.79811	0.00473	0.80284	0.00000
O 69	-0.61671	1.99977	6.60502	0.01193	8.61671	0.00000
C 70	-0.08855	1.99918	4.06980	0.01957	6.08855	0.00000
H 71	0.19753	0.00000	0.79771	0.00476	0.80247	0.00000
H 72	0.19946	0.00000	0.79456	0.00598	0.80054	0.00000
C 73	-0.08680	1.99918	4.06835	0.01927	6.08680	0.00002
H 74	0.19811	0.00000	0.79708	0.00480	0.80189	0.00000
H 75	0.19926	0.00000	0.79460	0.00613	0.80074	0.00000
C 76	-0.27244	1.99935	4.25768	0.01541	6.27244	0.00010
H 77	0.17953	0.00000	0.81663	0.00384	0.82047	0.00002
H 78	0.23144	0.00000	0.76562	0.00294	0.76856	0.00002
H 79	0.18620	0.00000	0.80983	0.00397	0.81380	0.00003
K 80	0.94552	17.98943	0.03048	0.03457	18.05448	0.00479
K 81	0.89222	17.98965	0.06316	0.05497	18.10778	0.00165
C 82	-0.38414	1.99900	4.36374	0.02140	6.38414	-0.04248
C 83	-0.30027	1.99897	4.28164	0.01966	6.30027	0.08795
C 84	-0.17717	1.99876	4.15687	0.02153	6.17717	-0.00505

C 85	-0.14879	1.99877	4.12858	0.02145	6.14879	0.04302
C 86	-0.44489	1.99902	4.42172	0.02415	6.44489	-0.00175
C 87	-0.12091	1.99876	4.10173	0.02042	6.12091	0.07496
C 88	-0.13003	1.99874	4.11104	0.02025	6.13003	0.05768
C 89	-0.16534	1.99874	4.14627	0.02033	6.16534	0.02754
C 90	-0.14994	1.99874	4.13035	0.02084	6.14994	0.00626
C 91	-0.12708	1.99876	4.10892	0.01941	6.12708	0.06912
Cs 92	0.92294	53.98872	0.04428	0.04406	54.07706	-0.00047
O 93	-0.61671	1.99977	6.60501	0.01193	8.61671	0.00000
O 94	-0.62585	1.99979	6.61513	0.01094	8.62585	0.00014
O 95	-0.62746	1.99977	6.61599	0.01170	8.62746	0.00009
O 96	-0.62804	1.99979	6.61737	0.01088	8.62804	0.00011
C 97	-0.32664	1.99900	4.30674	0.02091	6.32664	0.18039
H 98	0.22415	0.00000	0.77257	0.00328	0.77585	-0.00551
C 99	-0.40808	1.99902	4.38628	0.02278	6.40808	-0.01911
H100	0.22456	0.00000	0.77234	0.00310	0.77544	0.00053
C101	-0.11252	1.99876	4.09352	0.02023	6.11252	0.10651
H102	0.22155	0.00000	0.77531	0.00314	0.77845	0.00119
H103	0.21965	0.00000	0.77705	0.00330	0.78035	-0.00278
C104	-0.30192	1.99899	4.28285	0.02009	6.30192	0.12948
H105	0.22329	0.00000	0.77341	0.00330	0.77671	-0.00398
C106	-0.32989	1.99899	4.31038	0.02053	6.32989	0.08845
H107	0.22249	0.00000	0.77418	0.00333	0.77751	-0.00262
C108	-0.39065	1.99899	4.36926	0.02240	6.39065	0.03077
H109	0.22660	0.00000	0.77036	0.00304	0.77340	-0.00103
H110	0.22274	0.00000	0.77415	0.00311	0.77726	-0.00001
C111	-0.39237	1.99901	4.37058	0.02278	6.39237	0.04658
H112	0.22717	0.00000	0.76952	0.00331	0.77283	-0.00140
C113	-0.33077	1.99900	4.31045	0.02132	6.33077	0.19189
H114	0.22818	0.00000	0.76860	0.00323	0.77182	-0.00585
C115	-0.17985	1.99877	4.15969	0.02138	6.17985	-0.05900
C116	-0.16004	1.99873	4.14104	0.02027	6.16004	-0.00347
C117	-0.27570	1.99936	4.26038	0.01597	6.27570	0.00162
H118	0.20034	0.00000	0.79545	0.00421	0.79966	0.00072
H119	0.21797	0.00000	0.77795	0.00409	0.78203	0.00035
H120	0.18159	0.00000	0.81472	0.00368	0.81841	0.00011
C121	-0.08899	1.99917	4.07055	0.01927	6.08899	0.00018
H122	0.21315	0.00000	0.78084	0.00601	0.78685	-0.00004
H123	0.19786	0.00000	0.79774	0.00441	0.80214	0.00001
C124	-0.08335	1.99917	4.06493	0.01924	6.08335	0.00005
H125	0.19900	0.00000	0.79525	0.00575	0.80100	-0.00001
H126	0.19902	0.00000	0.79668	0.00431	0.80098	0.00001
C127	-0.08336	1.99917	4.06500	0.01919	6.08336	0.00011
H128	0.19861	0.00000	0.79712	0.00427	0.80139	0.00001
H129	0.19843	0.00000	0.79580	0.00576	0.80157	-0.00001
C130	-0.08891	1.99916	4.07037	0.01938	6.08891	0.00047
H131	0.19696	0.00000	0.79859	0.00445	0.80304	0.00000
H132	0.21497	0.00000	0.77913	0.00590	0.78503	0.00013
C133	-0.27858	1.99934	4.26325	0.01598	6.27858	0.00108
H134	0.17891	0.00000	0.81743	0.00365	0.82109	0.00013
H135	0.21814	0.00000	0.77758	0.00428	0.78186	0.00034
H136	0.20840	0.00000	0.78748	0.00412	0.79160	0.00005
O137	-0.61954	1.99978	6.60891	0.01085	8.61954	0.00001
O138	-0.61742	1.99978	6.60676	0.01088	8.61742	0.00001
C139	-0.27352	1.99934	4.25877	0.01542	6.27352	-0.00015
H140	0.18947	0.00000	0.80675	0.00378	0.81053	-0.00001
H141	0.23463	0.00000	0.76205	0.00332	0.76537	0.00002
H142	0.17773	0.00000	0.81846	0.00381	0.82227	0.00001
C143	-0.08655	1.99918	4.06805	0.01932	6.08655	0.00000
H144	0.19932	0.00000	0.79452	0.00616	0.80068	0.00000
H145	0.19802	0.00000	0.79716	0.00482	0.80198	0.00000
C146	-0.08882	1.99917	4.07009	0.01955	6.08882	0.00000
H147	0.19961	0.00000	0.79433	0.00606	0.80039	0.00000
H148	0.19715	0.00000	0.79811	0.00473	0.80285	0.00000
C149	-0.08855	1.99918	4.06980	0.01957	6.08855	0.00000
H150	0.19753	0.00000	0.79771	0.00476	0.80247	0.00000
H151	0.19946	0.00000	0.79456	0.00598	0.80054	0.00000
C152	-0.08681	1.99918	4.06835	0.01927	6.08681	0.00002
H153	0.19811	0.00000	0.79708	0.00480	0.80189	0.00000
H154	0.19927	0.00000	0.79460	0.00613	0.80073	0.00000
C155	-0.27243	1.99935	4.25767	0.01540	6.27243	0.00010

H156	0.17953	0.00000	0.81663	0.00384	0.82047	0.00002
H157	0.23143	0.00000	0.76563	0.00294	0.76857	0.00002
H158	0.18621	0.00000	0.80982	0.00397	0.81379	0.00003
* Total *	0.00000	331.86955	408.12525	2.00521	742.00000	2.00000

Table S11. NBO charges in **1H**-K-*full* system, optimized at the PBE0/TZVP/ZORA level of theory.

Atom No	Natural Charge	Natural Population				Natural Spin Density
		Core	Valence	Rydberg	Total	
Cs 1	0.92322	53.98828	0.04373	0.04478	54.07678	-0.00041
K 2	0.94522	17.99152	0.02847	0.03478	18.05478	0.00563
K 3	0.88728	17.98910	0.06718	0.05644	18.11272	0.00167
O 4	-0.62551	1.99980	6.61536	0.01035	8.62551	0.00019
O 5	-0.63012	1.99978	6.61920	0.01115	8.63012	0.00023
O 6	-0.61890	1.99980	6.60860	0.01051	8.61890	0.00020
C 7	-0.40728	1.99899	4.38459	0.02369	6.40728	0.05473
H 8	0.22633	0.00000	0.77060	0.00307	0.77367	-0.00171
C 9	-0.44986	1.99900	4.42552	0.02534	6.44986	-0.01299
H 10	0.22028	0.00000	0.77629	0.00343	0.77972	0.00025
C 11	-0.13450	1.99876	4.11492	0.02082	6.13450	0.07783
C 12	-0.41585	1.99899	4.39411	0.02276	6.41585	0.02797
H 13	0.21999	0.00000	0.77691	0.00310	0.78001	-0.00082
C 14	-0.30143	1.99897	4.28134	0.02112	6.30143	0.18885
H 15	0.22577	0.00000	0.77089	0.00334	0.77423	-0.00590
C 16	-0.18641	1.99874	4.16671	0.02095	6.18641	-0.05264
C 17	-0.28988	1.99898	4.27147	0.01943	6.28988	0.15807
H 18	0.22218	0.00000	0.77449	0.00332	0.77782	-0.00501
C 19	-0.35844	1.99900	4.33939	0.02005	6.35844	-0.03179
H 20	0.22171	0.00000	0.77508	0.00321	0.77829	0.00089
C 21	-0.11758	1.99877	4.09884	0.01998	6.11758	0.11299
C 22	-0.41842	1.99901	4.39645	0.02296	6.41842	-0.05156
H 23	0.22461	0.00000	0.77238	0.00301	0.77539	0.00149
C 24	-0.34364	1.99898	4.32339	0.02127	6.34364	0.10283
H 25	0.22575	0.00000	0.77102	0.00323	0.77425	-0.00322
C 26	-0.16385	1.99876	4.14375	0.02134	6.16385	-0.00742
C 27	-0.33361	1.99898	4.31280	0.02182	6.33361	0.13682
H 28	0.22966	0.00000	0.76714	0.00320	0.77034	-0.00411
C 29	-0.34017	1.99899	4.31942	0.02177	6.34017	0.11305
H 30	0.23016	0.00000	0.76622	0.00362	0.76984	-0.00323
C 31	-0.12479	1.99876	4.10618	0.01985	6.12479	0.03060
C 32	-0.10842	1.99870	4.08918	0.02054	6.10842	0.02052
C 33	-0.15423	1.99874	4.13556	0.01994	6.15423	0.00765
C 34	-0.12872	1.99875	4.11043	0.01955	6.12872	0.06522
C 35	-0.16013	1.99874	4.14080	0.02059	6.16013	-0.00491
C 36	-0.13998	1.99874	4.12100	0.02024	6.13998	0.06999
C 37	-0.28826	1.99932	4.27293	0.01600	6.28826	0.00165
H 38	0.21596	0.00000	0.77926	0.00479	0.78404	-0.00002
H 39	0.21626	0.00000	0.77867	0.00507	0.78374	0.00004
H 40	0.18071	0.00000	0.81567	0.00362	0.81929	0.00018
C 41	-0.09755	1.99915	4.07897	0.01943	6.09755	0.00039
H 42	0.21991	0.00000	0.77358	0.00651	0.78009	0.00002
H 43	0.19970	0.00000	0.79591	0.00439	0.80030	-0.00001
C 44	-0.08727	1.99916	4.06923	0.01888	6.08727	0.00017
H 45	0.20080	0.00000	0.79347	0.00573	0.79920	0.00001
H 46	0.20019	0.00000	0.79568	0.00413	0.79981	0.00000
C 47	-0.08916	1.99914	4.07131	0.01871	6.08916	0.00018
H 48	0.20348	0.00000	0.79237	0.00415	0.79652	0.00001
H 49	0.20484	0.00000	0.78951	0.00564	0.79516	-0.00002
C 50	-0.09682	1.99913	4.07834	0.01934	6.09682	0.00024
H 51	0.20065	0.00000	0.79503	0.00433	0.79935	0.00000
H 52	0.21473	0.00000	0.77904	0.00623	0.78527	0.00000
C 53	-0.28589	1.99936	4.27041	0.01612	6.28589	0.00180
H 54	0.18642	0.00000	0.80986	0.00372	0.81358	0.00021
H 55	0.21723	0.00000	0.77845	0.00432	0.78277	0.00019

H 56	0.19777	0.00000	0.79708	0.00515	0.80223	0.00109
O 57	-0.62413	1.99973	6.61286	0.01154	8.62413	0.00000
O 58	-0.62017	1.99979	6.60935	0.01103	8.62017	-0.00001
C 59	-0.25622	1.99935	4.23929	0.01757	6.25622	0.00080
H 60	0.17979	0.00000	0.81610	0.00411	0.82021	-0.00005
H 61	0.22933	0.00000	0.76711	0.00356	0.77067	0.00044
H 62	0.16948	0.00000	0.82636	0.00416	0.83052	-0.00006
C 63	-0.08629	1.99911	4.06785	0.01933	6.08629	0.00003
H 64	0.20475	0.00000	0.78958	0.00568	0.79525	0.00001
H 65	0.19859	0.00000	0.79673	0.00468	0.80141	0.00000
C 66	-0.11533	1.99914	4.09801	0.01818	6.11533	0.00000
H 67	0.20939	0.00000	0.78409	0.00652	0.79061	0.00000
H 68	0.21552	0.00000	0.78014	0.00434	0.78448	0.00000
O 69	-0.60851	1.99984	6.59832	0.01035	8.60851	0.00000
C 70	-0.11238	1.99919	4.09412	0.01907	6.11238	0.00001
H 71	0.20516	0.00000	0.79017	0.00467	0.79484	0.00000
H 72	0.20926	0.00000	0.78463	0.00611	0.79074	0.00000
C 73	-0.09230	1.99919	4.07490	0.01821	6.09230	0.00000
H 74	0.20125	0.00000	0.79422	0.00454	0.79875	0.00000
H 75	0.20715	0.00000	0.78730	0.00555	0.79285	0.00000
C 76	-0.27173	1.99935	4.25577	0.01660	6.27173	0.00053
H 77	0.17713	0.00000	0.81903	0.00384	0.82287	-0.00005
H 78	0.23341	0.00000	0.76317	0.00342	0.76659	0.00035
H 79	0.18270	0.00000	0.81331	0.00399	0.81730	-0.00005
K 80	0.94520	17.99152	0.02849	0.03479	18.05480	0.00563
K 81	0.88728	17.98910	0.06718	0.05644	18.11272	0.00167
C 82	-0.41583	1.99899	4.39405	0.02279	6.41583	0.02790
C 83	-0.30148	1.99897	4.28139	0.02112	6.30148	0.18877
C 84	-0.18643	1.99874	4.16673	0.02096	6.18643	-0.05264
C 85	-0.11760	1.99877	4.09885	0.01998	6.11760	0.11296
C 86	-0.34371	1.99898	4.32346	0.02127	6.34371	0.10267
C 87	-0.16379	1.99876	4.14369	0.02133	6.16379	-0.00732
C 88	-0.15422	1.99874	4.13555	0.01994	6.15422	0.00769
C 89	-0.12873	1.99875	4.11044	0.01955	6.12873	0.06522
C 90	-0.16018	1.99874	4.14085	0.02059	6.16018	-0.00494
C 91	-0.13992	1.99874	4.12095	0.02023	6.13992	0.07000
Cs 92	0.92320	53.98828	0.04373	0.04478	54.07680	-0.00041
O 93	-0.60850	1.99984	6.59831	0.01035	8.60850	0.00000
O 94	-0.62551	1.99980	6.61536	0.01035	8.62551	0.00019
O 95	-0.63012	1.99978	6.61920	0.01115	8.63012	0.00023
O 96	-0.61890	1.99980	6.60860	0.01051	8.61890	0.00020
C 97	-0.40724	1.99899	4.38457	0.02368	6.40724	0.05491
H 98	0.22631	0.00000	0.77062	0.00308	0.77369	-0.00172
C 99	-0.44958	1.99900	4.42521	0.02538	6.44958	-0.01310
H100	0.22024	0.00000	0.77633	0.00344	0.77976	0.00025
C101	-0.13441	1.99876	4.11481	0.02084	6.13441	0.07796
H102	0.21981	0.00000	0.77708	0.00311	0.78019	-0.00082
H103	0.22576	0.00000	0.77090	0.00334	0.77424	-0.00590
C104	-0.28987	1.99898	4.27147	0.01943	6.28987	0.15813
H105	0.22218	0.00000	0.77450	0.00333	0.77782	-0.00501
C106	-0.35838	1.99900	4.33934	0.02005	6.35838	-0.03172
H107	0.22171	0.00000	0.77508	0.00321	0.77829	0.00089
C108	-0.41845	1.99901	4.39647	0.02296	6.41845	-0.05157
H109	0.22462	0.00000	0.77237	0.00301	0.77538	0.00149
H110	0.22575	0.00000	0.77102	0.00323	0.77425	-0.00322
C111	-0.33366	1.99898	4.31285	0.02182	6.33366	0.13672
H112	0.22966	0.00000	0.76714	0.00320	0.77034	-0.00411
C113	-0.34014	1.99899	4.31938	0.02177	6.34014	0.11325
H114	0.23016	0.00000	0.76622	0.00362	0.76984	-0.00324
C115	-0.12483	1.99876	4.10622	0.01985	6.12483	0.03042
C116	-0.10845	1.99870	4.08921	0.02054	6.10845	0.02050
C117	-0.28826	1.99932	4.27293	0.01600	6.28826	0.00165
H118	0.21595	0.00000	0.77926	0.00479	0.78405	-0.00002
H119	0.21626	0.00000	0.77868	0.00507	0.78374	0.00004
H120	0.18071	0.00000	0.81567	0.00362	0.81929	0.00018
C121	-0.09754	1.99915	4.07897	0.01943	6.09754	0.00039
H122	0.21991	0.00000	0.77358	0.00651	0.78009	0.00002
H123	0.19970	0.00000	0.79591	0.00439	0.80030	-0.00001
C124	-0.08727	1.99916	4.06923	0.01888	6.08727	0.00017
H125	0.20080	0.00000	0.79347	0.00573	0.79920	0.00001
H126	0.20019	0.00000	0.79568	0.00413	0.79981	0.00000

C127	-0.08916	1.99914	4.07131	0.01871	6.08916	0.00018
H128	0.20348	0.00000	0.79237	0.00415	0.79652	0.00001
H129	0.20484	0.00000	0.78952	0.00564	0.79516	-0.00002
C130	-0.09682	1.99913	4.07835	0.01934	6.09682	0.00024
H131	0.20065	0.00000	0.79503	0.00433	0.79935	0.00000
H132	0.21475	0.00000	0.77902	0.00622	0.78525	0.00000
C133	-0.28589	1.99936	4.27041	0.01612	6.28589	0.00180
H134	0.18642	0.00000	0.80986	0.00372	0.81358	0.00021
H135	0.21722	0.00000	0.77845	0.00432	0.78278	0.00019
H136	0.19777	0.00000	0.79708	0.00515	0.80223	0.00109
O137	-0.62404	1.99973	6.61279	0.01152	8.62404	0.00000
O138	-0.62018	1.99979	6.60936	0.01103	8.62018	-0.00001
C139	-0.25631	1.99935	4.23939	0.01757	6.25631	0.00079
H140	0.18034	0.00000	0.81555	0.00411	0.81966	-0.00005
H141	0.22902	0.00000	0.76745	0.00353	0.77098	0.00042
H142	0.16935	0.00000	0.82650	0.00415	0.83065	-0.00006
C143	-0.08623	1.99911	4.06777	0.01935	6.08623	0.00002
H144	0.20463	0.00000	0.78968	0.00569	0.79537	0.00001
H145	0.19855	0.00000	0.79676	0.00468	0.80145	0.00000
C146	-0.11532	1.99914	4.09801	0.01817	6.11532	0.00000
H147	0.20936	0.00000	0.78411	0.00653	0.79064	0.00000
H148	0.21553	0.00000	0.78013	0.00434	0.78447	0.00000
C149	-0.11238	1.99919	4.09411	0.01907	6.11238	0.00001
H150	0.20516	0.00000	0.79017	0.00467	0.79484	0.00000
H151	0.20926	0.00000	0.78463	0.00611	0.79074	0.00000
C152	-0.09230	1.99919	4.07490	0.01821	6.09230	0.00000
H153	0.20125	0.00000	0.79421	0.00454	0.79875	0.00000
H154	0.20716	0.00000	0.78730	0.00554	0.79284	0.00000
C155	-0.27174	1.99935	4.25578	0.01660	6.27174	0.00053
H156	0.17716	0.00000	0.81900	0.00384	0.82284	-0.00005
H157	0.23342	0.00000	0.76316	0.00342	0.76658	0.00035
H158	0.18268	0.00000	0.81334	0.00399	0.81732	-0.00005
* Total *	0.00000	331.87110	408.11312	2.01578	742.00000	2.00000

Table S12. NBO charges in **1H**-K-*small* system, optimized at the PBE0/TZVP/ZORA level of theory.

Atom No	Natural Charge	Natural Population Core	Valence	Rydberg	Total	Natural Spin Density
Cs 1	0.96695	53.99326	0.00857	0.03122	54.03305	-0.00046
K 2	0.95722	17.99361	0.01514	0.03404	18.04278	0.00366
K 3	0.95692	17.99370	0.01661	0.03278	18.04308	0.00634
C 4	-0.40124	1.99899	4.37916	0.02309	6.40124	0.06936
H 5	0.22967	0.00000	0.76720	0.00314	0.77033	-0.00217
C 6	-0.44785	1.99900	4.42366	0.02519	6.44785	-0.01386
H 7	0.22860	0.00000	0.76821	0.00319	0.77140	0.00030
C 8	-0.14278	1.99877	4.12262	0.02139	6.14278	0.08304
C 9	-0.40636	1.99900	4.38391	0.02346	6.40636	0.02912
H 10	0.23134	0.00000	0.76544	0.00322	0.76866	-0.00080
C 11	-0.32636	1.99900	4.30628	0.02109	6.32636	0.17353
H 12	0.23158	0.00000	0.76522	0.00320	0.76842	-0.00538
C 13	-0.19975	1.99876	4.17981	0.02118	6.19975	-0.04761
C 14	-0.26846	1.99898	4.25085	0.01864	6.26846	0.14456
H 15	0.22590	0.00000	0.77079	0.00331	0.77410	-0.00465
C 16	-0.33647	1.99900	4.31802	0.01945	6.33647	-0.04255
H 17	0.22617	0.00000	0.77058	0.00325	0.77383	0.00122
C 18	-0.11687	1.99877	4.09821	0.01988	6.11687	0.12186
C 19	-0.40294	1.99901	4.38134	0.02259	6.40294	-0.06103
H 20	0.23073	0.00000	0.76614	0.00314	0.76927	0.00183
C 21	-0.33348	1.99899	4.31373	0.02076	6.33348	0.11537
H 22	0.22820	0.00000	0.76859	0.00322	0.77180	-0.00367
C 23	-0.17489	1.99878	4.15418	0.02193	6.17489	-0.02397
C 24	-0.37242	1.99898	4.34959	0.02384	6.37242	0.16039
H 25	0.22675	0.00000	0.76988	0.00337	0.77325	-0.00454
C 26	-0.36813	1.99899	4.34535	0.02379	6.36813	0.13906

H	27	0.22705	0.00000	0.76954	0.00341	0.77295	-0.00386
C	28	-0.12829	1.99877	4.10905	0.02047	6.12829	0.01844
C	29	-0.11890	1.99871	4.09936	0.02084	6.11890	0.02317
C	30	-0.16379	1.99874	4.14504	0.02000	6.16379	0.01407
C	31	-0.12960	1.99875	4.11163	0.01921	6.12960	0.04766
C	32	-0.17579	1.99875	4.15603	0.02101	6.17579	-0.00205
C	33	-0.15267	1.99876	4.13368	0.02022	6.15267	0.06361
K	34	0.95722	17.99361	0.01514	0.03404	18.04278	0.00366
K	35	0.95692	17.99370	0.01661	0.03278	18.04308	0.00634
C	36	-0.40636	1.99900	4.38391	0.02346	6.40636	0.02912
C	37	-0.32636	1.99900	4.30628	0.02109	6.32636	0.17353
C	38	-0.19975	1.99876	4.17981	0.02118	6.19975	-0.04761
C	39	-0.11687	1.99877	4.09821	0.01988	6.11687	0.12186
C	40	-0.33349	1.99899	4.31373	0.02076	6.33349	0.11537
C	41	-0.17489	1.99878	4.15418	0.02193	6.17489	-0.02397
C	42	-0.16379	1.99874	4.14505	0.02000	6.16379	0.01407
C	43	-0.12960	1.99875	4.11163	0.01921	6.12960	0.04766
C	44	-0.17579	1.99875	4.15604	0.02101	6.17579	-0.00205
C	45	-0.15267	1.99876	4.13368	0.02022	6.15267	0.06361
Cs	46	0.96695	53.99326	0.00857	0.03122	54.03305	-0.00046
C	47	-0.40124	1.99899	4.37916	0.02309	6.40124	0.06936
H	48	0.22966	0.00000	0.76720	0.00314	0.77034	-0.00217
C	49	-0.44785	1.99900	4.42366	0.02519	6.44785	-0.01386
H	50	0.22860	0.00000	0.76821	0.00319	0.77140	0.00030
C	51	-0.14278	1.99877	4.12261	0.02139	6.14278	0.08304
H	52	0.23134	0.00000	0.76544	0.00322	0.76866	-0.00080
H	53	0.23158	0.00000	0.76522	0.00320	0.76842	-0.00538
C	54	-0.26846	1.99898	4.25085	0.01864	6.26846	0.14456
H	55	0.22590	0.00000	0.77079	0.00331	0.77410	-0.00465
C	56	-0.33647	1.99900	4.31802	0.01945	6.33647	-0.04255
H	57	0.22617	0.00000	0.77058	0.00325	0.77383	0.00122
C	58	-0.40294	1.99901	4.38134	0.02259	6.40294	-0.06103
H	59	0.23073	0.00000	0.76614	0.00314	0.76927	0.00183
H	60	0.22820	0.00000	0.76859	0.00322	0.77180	-0.00367
C	61	-0.37242	1.99898	4.34959	0.02384	6.37242	0.16039
H	62	0.22675	0.00000	0.76988	0.00337	0.77325	-0.00454
C	63	-0.36813	1.99899	4.34534	0.02379	6.36813	0.13907
H	64	0.22705	0.00000	0.76954	0.00341	0.77295	-0.00386
C	65	-0.12829	1.99877	4.10905	0.02047	6.12829	0.01844
C	66	-0.11890	1.99871	4.09936	0.02084	6.11890	0.02317
* Total *		0.00000	259.91619	184.96680	1.11701	446.00000	2.00000

Table S13. NBO charges in **1**-Li-*small* system, optimized at the PBE0/def2-TZVP(Li,Cs)//cc-pVDZ(C,H,O) level of theory.

Atom	No	Natural Charge	Natural Population				Natural Spin Density
			Core	Valence	Rydberg	Total	
Cs	1	0.96335	53.99264	0.00783	0.03618	54.03665	0.00074
Li	2	0.92480	1.99788	0.05405	0.02327	2.07520	0.00127
C	3	-0.40101	1.99903	4.37872	0.02326	6.40101	-0.01166
H	4	0.24122	0.00000	0.75572	0.00306	0.75878	0.00030
C	5	-0.36597	1.99901	4.34511	0.02185	6.36597	0.02739
H	6	0.24292	0.00000	0.75410	0.00298	0.75708	-0.00086
C	7	-0.16401	1.99875	4.14219	0.02307	6.16401	0.04234
C	8	-0.25300	1.99900	4.23536	0.01864	6.25300	0.06914
H	9	0.23153	0.00000	0.76524	0.00323	0.76847	-0.00219
C	10	-0.25329	1.99901	4.23563	0.01866	6.25329	0.06886
H	11	0.23149	0.00000	0.76527	0.00324	0.76851	-0.00218
C	12	-0.16548	1.99876	4.14362	0.02310	6.16548	0.04227
C	13	-0.36863	1.99901	4.34770	0.02192	6.36863	0.02842
H	14	0.24313	0.00000	0.75390	0.00297	0.75687	-0.00089
C	15	-0.40448	1.99903	4.38203	0.02343	6.40448	-0.01240
H	16	0.24131	0.00000	0.75563	0.00306	0.75869	0.00032
C	17	-0.12121	1.99877	4.09889	0.02355	6.12121	0.07801
C	18	-0.49122	1.99905	4.46567	0.02650	6.49122	0.02943

H	19	0.24529	0.00000	0.75175	0.00296	0.75471	-0.00091
C	20	-0.34306	1.99903	4.32142	0.02261	6.34306	0.24556
H	21	0.24073	0.00000	0.75617	0.00310	0.75927	-0.00728
C	22	-0.15340	1.99875	4.13318	0.02147	6.15340	-0.08471
C	23	-0.33853	1.99903	4.31695	0.02256	6.33853	0.24611
H	24	0.24023	0.00000	0.75666	0.00311	0.75977	-0.00729
C	25	-0.49177	1.99906	4.46623	0.02649	6.49177	0.03096
H	26	0.24508	0.00000	0.75195	0.00297	0.75492	-0.00094
C	27	-0.11918	1.99877	4.09688	0.02353	6.11918	0.07669
C	28	-0.14266	1.99879	4.12469	0.01918	6.14266	0.06708
C	29	-0.16682	1.99878	4.14753	0.02051	6.16682	0.00466
C	30	-0.16864	1.99878	4.14924	0.02063	6.16864	0.00381
C	31	-0.14496	1.99880	4.12701	0.01916	6.14496	0.06844
C	32	-0.15818	1.99877	4.13394	0.02547	6.15818	-0.00184
Li	33	0.92443	1.99786	0.05438	0.02332	2.07557	0.00138
Cs	34	0.96334	53.99264	0.00783	0.03619	54.03666	0.00074
Li	35	0.92481	1.99788	0.05404	0.02327	2.07519	0.00126
C	36	-0.40096	1.99903	4.37867	0.02325	6.40096	-0.01143
H	37	0.24123	0.00000	0.75571	0.00306	0.75877	0.00029
C	38	-0.36622	1.99901	4.34535	0.02185	6.36622	0.02700
H	39	0.24291	0.00000	0.75411	0.00298	0.75709	-0.00085
C	40	-0.16379	1.99875	4.14197	0.02306	6.16379	0.04268
C	41	-0.25314	1.99900	4.23549	0.01865	6.25314	0.06880
H	42	0.23153	0.00000	0.76524	0.00323	0.76847	-0.00218
C	43	-0.25315	1.99900	4.23549	0.01866	6.25315	0.06917
H	44	0.23150	0.00000	0.76526	0.00324	0.76850	-0.00219
C	45	-0.16566	1.99876	4.14380	0.02311	6.16566	0.04194
C	46	-0.36835	1.99901	4.34743	0.02191	6.36835	0.02881
H	47	0.24311	0.00000	0.75391	0.00297	0.75689	-0.00090
C	48	-0.40455	1.99903	4.38209	0.02343	6.40455	-0.01261
H	49	0.24130	0.00000	0.75563	0.00306	0.75870	0.00033
C	50	-0.12105	1.99877	4.09874	0.02355	6.12105	0.07820
C	51	-0.49127	1.99905	4.46571	0.02650	6.49127	0.02910
H	52	0.24529	0.00000	0.75175	0.00296	0.75471	-0.00090
C	53	-0.34331	1.99903	4.32167	0.02261	6.34331	0.24529
H	54	0.24073	0.00000	0.75617	0.00310	0.75927	-0.00727
C	55	-0.15338	1.99875	4.13316	0.02147	6.15338	-0.08471
C	56	-0.33834	1.99903	4.31676	0.02255	6.33834	0.24639
H	57	0.24021	0.00000	0.75668	0.00311	0.75979	-0.00730
C	58	-0.49172	1.99906	4.46618	0.02649	6.49172	0.03133
H	59	0.24509	0.00000	0.75194	0.00297	0.75491	-0.00095
C	60	-0.11933	1.99877	4.09703	0.02353	6.11933	0.07647
C	61	-0.14276	1.99879	4.12478	0.01918	6.14276	0.06697
C	62	-0.16673	1.99878	4.14744	0.02051	6.16673	0.00478
C	63	-0.16872	1.99878	4.14931	0.02063	6.16872	0.00367
C	64	-0.14488	1.99880	4.12693	0.01916	6.14488	0.06854
C	65	-0.15819	1.99877	4.13394	0.02548	6.15819	-0.00184
Li	66	0.92444	1.99786	0.05438	0.02333	2.07556	0.00138
* Total *		0.00000	195.93271	184.94919	1.11810	382.00000	2.00000

Table S14. NBO charges in **1**-Na-*small* system, optimized at the PBE0/def2-TZVP(Na,Cs)//cc-pVDZ(C,H,O) level of theory.

Atom	No	Natural Charge	Natural Population: Core	Valence	Rydberg	Total	Natural Spin Density
Cs	1	0.96650	53.99115	0.00890	0.03344	54.03350	0.00057
Na	2	0.95233	9.99614	0.03098	0.02055	10.04767	0.00107
C	3	-0.32750	1.99901	4.30745	0.02104	6.32750	0.12684
H	4	0.23512	0.00000	0.76163	0.00325	0.76488	-0.00374
C	5	-0.33540	1.99901	4.31600	0.02039	6.33540	0.11897
H	6	0.23681	0.00000	0.76004	0.00316	0.76319	-0.00359
C	7	-0.18254	1.99878	4.16274	0.02102	6.18254	0.00166
C	8	-0.28032	1.99899	4.26231	0.01902	6.28032	0.08421
H	9	0.22975	0.00000	0.76702	0.00322	0.77025	-0.00266

C	10	-0.32576	1.99900	4.30702	0.01974	6.32576	-0.04866
H	11	0.23019	0.00000	0.76661	0.00319	0.76981	0.00142
C	12	-0.14428	1.99878	4.12471	0.02079	6.14428	0.11682
C	13	-0.38522	1.99902	4.36492	0.02127	6.38522	-0.02271
H	14	0.23585	0.00000	0.76103	0.00311	0.76415	0.00074
C	15	-0.27615	1.99900	4.25743	0.01973	6.27615	0.18211
H	16	0.23302	0.00000	0.76372	0.00327	0.76698	-0.00569
C	17	-0.20781	1.99879	4.18740	0.02161	6.20781	-0.05868
C	18	-0.35223	1.99901	4.33199	0.02123	6.35223	0.18096
H	19	0.23838	0.00000	0.75853	0.00308	0.76162	-0.00554
C	20	-0.43179	1.99903	4.40885	0.02392	6.43179	0.03022
H	21	0.23784	0.00000	0.75903	0.00313	0.76216	-0.00089
C	22	-0.11993	1.99877	4.09994	0.02122	6.11993	0.09161
C	23	-0.44652	1.99902	4.42379	0.02371	6.44652	-0.01990
H	24	0.23748	0.00000	0.75944	0.00307	0.76252	0.00046
C	25	-0.41283	1.99901	4.39117	0.02265	6.41283	0.07533
H	26	0.23845	0.00000	0.75855	0.00301	0.76155	-0.00233
C	27	-0.16414	1.99878	4.14387	0.02149	6.16414	0.02001
C	28	-0.18111	1.99876	4.16231	0.02004	6.18111	0.01003
C	29	-0.16726	1.99877	4.14875	0.01974	6.16726	0.01763
C	30	-0.15388	1.99878	4.13568	0.01943	6.15388	0.04572
C	31	-0.19078	1.99876	4.17185	0.02017	6.19078	-0.00418
C	32	-0.13980	1.99875	4.12040	0.02065	6.13980	0.07001
Na	33	0.95355	9.99624	0.02977	0.02044	10.04645	0.00218
Cs	34	0.96649	53.99116	0.00891	0.03345	54.03351	0.00056
Na	35	0.95229	9.99614	0.03104	0.02054	10.04771	0.00106
C	36	-0.32826	1.99901	4.30820	0.02105	6.32826	0.12506
H	37	0.23512	0.00000	0.76163	0.00325	0.76488	-0.00369
C	38	-0.33453	1.99901	4.31514	0.02037	6.33453	0.12013
H	39	0.23679	0.00000	0.76005	0.00316	0.76321	-0.00363
C	40	-0.18321	1.99878	4.16340	0.02103	6.18321	0.00030
C	41	-0.27956	1.99899	4.26156	0.01901	6.27956	0.08591
H	42	0.22976	0.00000	0.76702	0.00322	0.77024	-0.00271
C	43	-0.32602	1.99900	4.30728	0.01974	6.32602	-0.04966
H	44	0.23020	0.00000	0.76661	0.00319	0.76980	0.00145
C	45	-0.14384	1.99878	4.12428	0.02079	6.14384	0.11784
C	46	-0.38593	1.99902	4.36562	0.02129	6.38593	-0.02433
H	47	0.23587	0.00000	0.76102	0.00311	0.76413	0.00079
C	48	-0.27630	1.99899	4.25758	0.01973	6.27630	0.18195
H	49	0.23301	0.00000	0.76372	0.00327	0.76699	-0.00569
C	50	-0.20794	1.99879	4.18754	0.02161	6.20794	-0.05899
C	51	-0.35222	1.99901	4.33197	0.02124	6.35222	0.18168
H	52	0.23841	0.00000	0.75851	0.00308	0.76159	-0.00556
C	53	-0.43115	1.99903	4.40821	0.02391	6.43115	0.03238
H	54	0.23785	0.00000	0.75902	0.00313	0.76215	-0.00095
C	55	-0.12037	1.99877	4.10038	0.02122	6.12037	0.09062
C	56	-0.44603	1.99902	4.42332	0.02369	6.44603	-0.01895
H	57	0.23748	0.00000	0.75945	0.00307	0.76252	0.00043
C	58	-0.41390	1.99901	4.39222	0.02267	6.41390	0.07320
H	59	0.23846	0.00000	0.75853	0.00301	0.76154	-0.00226
C	60	-0.16324	1.99878	4.14298	0.02148	6.16324	0.02164
C	61	-0.18067	1.99876	4.16188	0.02003	6.18067	0.01056
C	62	-0.16749	1.99877	4.14897	0.01975	6.16749	0.01717
C	63	-0.15364	1.99878	4.13544	0.01942	6.15364	0.04593
C	64	-0.19079	1.99876	4.17186	0.02017	6.19079	-0.00395
C	65	-0.14018	1.99875	4.12076	0.02066	6.14018	0.06949
Na	66	0.95351	9.99624	0.02980	0.02045	10.04649	0.00218
* Total *		0.00000	227.92267	185.02773	1.04959	414.00000	2.00000

Table S15. NBO charges in **1**-K-*small* system, optimized at the PBE0/def2-TZVP(K,Cs)//cc-pVDZ(C,H,O) level of theory.

Atom	No	Natural Charge	Natural Population: Core	Valence	Rydberg	Total	Natural Spin Density
Cs	1	0.96561	53.99185	0.00993	0.03261	54.03439	-0.00034

K	2	0.95683	17.99150	0.02074	0.03092	18.04317	0.00523
C	3	-0.34578	1.99900	4.32495	0.02183	6.34578	0.10008
H	4	0.23127	0.00000	0.76549	0.00323	0.76873	-0.00283
C	5	-0.32845	1.99900	4.30849	0.02096	6.32845	0.14330
H	6	0.23204	0.00000	0.76477	0.00320	0.76796	-0.00436
C	7	-0.19417	1.99878	4.17367	0.02171	6.19417	-0.02055
C	8	-0.28264	1.99898	4.26450	0.01915	6.28264	0.11377
H	9	0.22719	0.00000	0.76955	0.00325	0.77281	-0.00361
C	10	-0.35255	1.99900	4.33299	0.02057	6.35255	-0.05872
H	11	0.22798	0.00000	0.76885	0.00317	0.77202	0.00177
C	12	-0.13897	1.99878	4.11915	0.02105	6.13897	0.12790
C	13	-0.39043	1.99901	4.36920	0.02222	6.39043	-0.04721
H	14	0.23059	0.00000	0.76626	0.00315	0.76941	0.00145
C	15	-0.27932	1.99899	4.26073	0.01960	6.27932	0.16550
H	16	0.22840	0.00000	0.76831	0.00329	0.77160	-0.00523
C	17	-0.20796	1.99879	4.18709	0.02208	6.20796	-0.05373
C	18	-0.33768	1.99900	4.31726	0.02142	6.33768	0.18155
H	19	0.23258	0.00000	0.76423	0.00319	0.76742	-0.00555
C	20	-0.40189	1.99901	4.37896	0.02392	6.40189	0.05782
H	21	0.23270	0.00000	0.76411	0.00319	0.76730	-0.00161
C	22	-0.13262	1.99878	4.11259	0.02126	6.13262	0.07564
C	23	-0.42674	1.99900	4.40384	0.02391	6.42674	-0.00094
H	24	0.23265	0.00000	0.76424	0.00311	0.76735	-0.00008
C	25	-0.41110	1.99899	4.38873	0.02337	6.41110	0.04458
H	26	0.23282	0.00000	0.76408	0.00311	0.76718	-0.00142
C	27	-0.15241	1.99878	4.13218	0.02145	6.15241	0.04194
C	28	-0.17001	1.99875	4.15032	0.02095	6.17001	0.02498
C	29	-0.16551	1.99875	4.14607	0.02068	6.16551	0.01018
C	30	-0.13962	1.99876	4.12096	0.01990	6.13962	0.04902
C	31	-0.18067	1.99874	4.16043	0.02149	6.18067	0.00058
C	32	-0.15203	1.99874	4.13247	0.02082	6.15203	0.05539
K	33	0.95991	17.99195	0.01785	0.03029	18.04009	0.00550
Cs	34	0.96561	53.99185	0.00993	0.03261	54.03439	-0.00034
K	35	0.95683	17.99150	0.02074	0.03093	18.04317	0.00523
C	36	-0.34576	1.99900	4.32492	0.02183	6.34576	0.10010
H	37	0.23127	0.00000	0.76550	0.00323	0.76873	-0.00283
C	38	-0.32846	1.99900	4.30849	0.02096	6.32846	0.14330
H	39	0.23203	0.00000	0.76477	0.00320	0.76797	-0.00436
C	40	-0.19415	1.99878	4.17366	0.02171	6.19415	-0.02053
C	41	-0.28265	1.99898	4.26451	0.01915	6.28265	0.11373
H	42	0.22719	0.00000	0.76955	0.00325	0.77281	-0.00361
C	43	-0.35255	1.99900	4.33299	0.02057	6.35255	-0.05871
H	44	0.22798	0.00000	0.76885	0.00317	0.77202	0.00177
C	45	-0.13897	1.99878	4.11914	0.02105	6.13897	0.12788
C	46	-0.39042	1.99901	4.36919	0.02222	6.39042	-0.04720
H	47	0.23059	0.00000	0.76626	0.00315	0.76941	0.00144
C	48	-0.27931	1.99899	4.26073	0.01960	6.27931	0.16552
H	49	0.22840	0.00000	0.76831	0.00329	0.77160	-0.00523
C	50	-0.20796	1.99879	4.18709	0.02208	6.20796	-0.05374
C	51	-0.33768	1.99900	4.31726	0.02142	6.33768	0.18156
H	52	0.23258	0.00000	0.76423	0.00319	0.76742	-0.00555
C	53	-0.40191	1.99901	4.37898	0.02392	6.40191	0.05778
H	54	0.23269	0.00000	0.76412	0.00319	0.76731	-0.00161
C	55	-0.13261	1.99878	4.11257	0.02126	6.13261	0.07567
C	56	-0.42675	1.99900	4.40384	0.02391	6.42675	-0.00096
H	57	0.23265	0.00000	0.76425	0.00311	0.76735	-0.00008
C	58	-0.41108	1.99899	4.38871	0.02337	6.41108	0.04462
H	59	0.23282	0.00000	0.76408	0.00311	0.76718	-0.00142
C	60	-0.15243	1.99878	4.13220	0.02145	6.15243	0.04193
C	61	-0.17004	1.99875	4.15034	0.02095	6.17004	0.02497
C	62	-0.16550	1.99875	4.14607	0.02068	6.16550	0.01019
C	63	-0.13963	1.99876	4.12097	0.01990	6.13963	0.04901
C	64	-0.18067	1.99874	4.16043	0.02149	6.18067	0.00057
C	65	-0.15203	1.99874	4.13247	0.02082	6.15203	0.05539
K	66	0.95991	17.99195	0.01785	0.03029	18.04009	0.00550
* Total *		0.00000	259.90589	184.98601	1.10810	446.00000	2.00000

Table S16. NBO charges in **1**-Rb-*small* system, optimized at the PBE0/def2-TZVP(Rb,Cs)//cc-pVDZ(C,H,O) level of theory.

Atom	No	Natural Charge	Natural Population: Core	Valence	Rydberg	Total	Natural Spin Density
Cs	1	0.96443	53.99204	0.01072	0.03281	54.03557	-0.00027
Rb	2	0.95380	35.99023	0.02219	0.03379	36.04620	0.00752
C	3	-0.35334	1.99900	4.33221	0.02213	6.35334	0.08607
H	4	0.23000	0.00000	0.76673	0.00327	0.77000	-0.00238
C	5	-0.32348	1.99900	4.30361	0.02088	6.32348	0.14960
H	6	0.23019	0.00000	0.76655	0.00326	0.76981	-0.00456
C	7	-0.19782	1.99879	4.17750	0.02153	6.19782	-0.02758
C	8	-0.28527	1.99898	4.26688	0.01941	6.28527	0.12487
H	9	0.22653	0.00000	0.77017	0.00330	0.77347	-0.00394
C	10	-0.36254	1.99900	4.34245	0.02109	6.36254	-0.05811
H	11	0.22728	0.00000	0.76949	0.00323	0.77272	0.00177
C	12	-0.13650	1.99878	4.11694	0.02077	6.13650	0.13002
C	13	-0.39299	1.99901	4.37158	0.02241	6.39299	-0.05272
H	14	0.22901	0.00000	0.76778	0.00321	0.77099	0.00161
C	15	-0.28695	1.99898	4.26818	0.01979	6.28695	0.15675
H	16	0.22725	0.00000	0.76943	0.00332	0.77275	-0.00494
C	17	-0.20235	1.99879	4.18180	0.02175	6.20235	-0.04822
C	18	-0.33564	1.99900	4.31528	0.02136	6.33564	0.17736
H	19	0.23058	0.00000	0.76614	0.00327	0.76942	-0.00541
C	20	-0.39200	1.99901	4.36926	0.02373	6.39200	0.07224
H	21	0.23113	0.00000	0.76561	0.00326	0.76887	-0.00201
C	22	-0.13484	1.99878	4.11507	0.02100	6.13484	0.06458
C	23	-0.41531	1.99899	4.39269	0.02363	6.41531	0.01103
H	24	0.23074	0.00000	0.76608	0.00318	0.76926	-0.00043
C	25	-0.40998	1.99899	4.38770	0.02329	6.40998	0.02887
H	26	0.23078	0.00000	0.76602	0.00320	0.76922	-0.00095
C	27	-0.14580	1.99878	4.12593	0.02110	6.14580	0.05170
C	28	-0.16196	1.99874	4.14225	0.02097	6.16196	0.03130
C	29	-0.16764	1.99875	4.14782	0.02107	6.16764	0.00654
C	30	-0.13703	1.99876	4.11819	0.02009	6.13703	0.05106
C	31	-0.17570	1.99874	4.15537	0.02159	6.17570	0.00299
C	32	-0.15259	1.99874	4.13294	0.02091	6.15259	0.04791
Rb	33	0.95802	35.99079	0.01788	0.03330	36.04198	0.00773
Cs	34	0.96443	53.99204	0.01072	0.03281	54.03557	-0.00027
Rb	35	0.95379	35.99023	0.02219	0.03379	36.04621	0.00751
C	36	-0.35328	1.99900	4.33215	0.02212	6.35328	0.08621
H	37	0.22999	0.00000	0.76674	0.00327	0.77001	-0.00239
C	38	-0.32351	1.99900	4.30364	0.02088	6.32351	0.14954
H	39	0.23019	0.00000	0.76655	0.00326	0.76981	-0.00456
C	40	-0.19779	1.99879	4.17747	0.02153	6.19779	-0.02751
C	41	-0.28532	1.99898	4.26693	0.01941	6.28532	0.12476
H	42	0.22653	0.00000	0.77017	0.00330	0.77347	-0.00393
C	43	-0.36255	1.99900	4.34246	0.02109	6.36255	-0.05812
H	44	0.22728	0.00000	0.76949	0.00323	0.77272	0.00177
C	45	-0.13650	1.99878	4.11695	0.02077	6.13650	0.13001
C	46	-0.39298	1.99901	4.37156	0.02241	6.39298	-0.05267
H	47	0.22901	0.00000	0.76778	0.00321	0.77099	0.00160
C	48	-0.28692	1.99898	4.26814	0.01979	6.28692	0.15687
H	49	0.22725	0.00000	0.76943	0.00332	0.77275	-0.00495
C	50	-0.20237	1.99879	4.18182	0.02175	6.20237	-0.04828
C	51	-0.33561	1.99900	4.31525	0.02136	6.33561	0.17738
H	52	0.23058	0.00000	0.76615	0.00327	0.76942	-0.00541
C	53	-0.39206	1.99901	4.36932	0.02373	6.39206	0.07209
H	54	0.23113	0.00000	0.76561	0.00326	0.76887	-0.00201
C	55	-0.13479	1.99878	4.11502	0.02099	6.13479	0.06467
C	56	-0.41537	1.99899	4.39274	0.02363	6.41537	0.01090
H	57	0.23073	0.00000	0.76609	0.00318	0.76927	-0.00043
C	58	-0.40990	1.99899	4.38762	0.02329	6.40990	0.02901
H	59	0.23078	0.00000	0.76603	0.00320	0.76922	-0.00096
C	60	-0.14585	1.99878	4.12598	0.02110	6.14585	0.05159
C	61	-0.16199	1.99874	4.14227	0.02098	6.16199	0.03124
C	62	-0.16764	1.99875	4.14782	0.02107	6.16764	0.00657

C 63	-0.13703	1.99876	4.11819	0.02009	6.13703	0.05105
C 64	-0.17573	1.99874	4.15539	0.02159	6.17573	0.00296
C 65	-0.15254	1.99874	4.13290	0.02091	6.15254	0.04797
Rb 66	0.95802	35.99079	0.01788	0.03330	36.04198	0.00773
* Total *	0.00000	331.90132	184.97689	1.12180	518.00000	2.00000

Table S17. NBO charges in **1**-Cs-*small* system, optimized at the PBE0/def2-TZVP(Cs)//cc-pVDZ(C,H,O) level of theory.

Atom No	Natural Charge	Natural Population				Natural Spin Density
		Core	Valence	Rydberg	Total	
Cs 1	0.96104	53.99240	0.01207	0.03448	54.03896	-0.00016
Cs 2	0.92193	53.98571	0.03688	0.05547	54.07807	0.01010
C 3	-0.36948	1.99900	4.34787	0.02262	6.36948	0.02367
H 4	0.22889	0.00000	0.76789	0.00323	0.77111	-0.00055
C 5	-0.31008	1.99898	4.29005	0.02105	6.31008	0.14874
H 6	0.22834	0.00000	0.76830	0.00336	0.77166	-0.00458
C 7	-0.21318	1.99879	4.19136	0.02304	6.21318	-0.04043
C 8	-0.27686	1.99898	4.25828	0.01961	6.27686	0.14952
H 9	0.22619	0.00000	0.77049	0.00331	0.77381	-0.00467
C 10	-0.35730	1.99899	4.33690	0.02141	6.35730	-0.02901
H 11	0.22698	0.00000	0.76976	0.00325	0.77302	0.00096
C 12	-0.14172	1.99878	4.12107	0.02186	6.14172	0.11888
C 13	-0.38689	1.99899	4.36512	0.02277	6.38689	-0.05061
H 14	0.22750	0.00000	0.76917	0.00334	0.77250	0.00150
C 15	-0.31194	1.99898	4.29243	0.02053	6.31194	0.10717
H 16	0.22675	0.00000	0.76998	0.00327	0.77325	-0.00339
C 17	-0.17909	1.99878	4.15797	0.02233	6.17909	-0.00931
C 18	-0.34952	1.99900	4.32792	0.02260	6.34952	0.14144
H 19	0.22984	0.00000	0.76672	0.00344	0.77016	-0.00425
C 20	-0.35500	1.99900	4.33303	0.02297	6.35500	0.13702
H 21	0.22974	0.00000	0.76690	0.00336	0.77026	-0.00399
C 22	-0.14624	1.99877	4.12616	0.02131	6.14624	0.01371
C 23	-0.37646	1.99898	4.35441	0.02307	6.37646	0.07835
H 24	0.22936	0.00000	0.76735	0.00329	0.77064	-0.00244
C 25	-0.41780	1.99899	4.39455	0.02425	6.41780	-0.01813
H 26	0.22981	0.00000	0.76680	0.00340	0.77019	0.00041
C 27	-0.13068	1.99877	4.11051	0.02139	6.13068	0.08874
C 28	-0.14488	1.99874	4.12499	0.02115	6.14488	0.05415
C 29	-0.17336	1.99873	4.15210	0.02253	6.17336	-0.00324
C 30	-0.13495	1.99874	4.11522	0.02098	6.13495	0.05007
C 31	-0.16078	1.99873	4.14027	0.02178	6.16078	0.01649
C 32	-0.15773	1.99873	4.13764	0.02135	6.15773	0.02289
Cs 33	0.92756	53.98666	0.03086	0.05492	54.07244	0.01095
Cs 34	0.96104	53.99240	0.01208	0.03448	54.03896	-0.00016
Cs 35	0.92193	53.98571	0.03688	0.05548	54.07807	0.01011
C 36	-0.36960	1.99900	4.34798	0.02262	6.36960	0.02322
H 37	0.22888	0.00000	0.76790	0.00323	0.77112	-0.00054
C 38	-0.31008	1.99898	4.29005	0.02105	6.31008	0.14877
H 39	0.22834	0.00000	0.76831	0.00335	0.77166	-0.00458
C 40	-0.21316	1.99879	4.19133	0.02304	6.21316	-0.04048
C 41	-0.27680	1.99898	4.25822	0.01961	6.27680	0.14964
H 42	0.22619	0.00000	0.77049	0.00331	0.77381	-0.00467
C 43	-0.35723	1.99899	4.33683	0.02140	6.35723	-0.02872
H 44	0.22700	0.00000	0.76975	0.00325	0.77300	0.00096
C 45	-0.14175	1.99878	4.12110	0.02186	6.14175	0.11872
C 46	-0.38685	1.99899	4.36509	0.02277	6.38685	-0.05047
H 47	0.22751	0.00000	0.76916	0.00334	0.77249	0.00150
C 48	-0.31212	1.99898	4.29261	0.02053	6.31212	0.10676
H 49	0.22675	0.00000	0.76998	0.00327	0.77325	-0.00338
C 50	-0.17894	1.99878	4.15783	0.02233	6.17894	-0.00902
C 51	-0.34969	1.99900	4.32809	0.02260	6.34969	0.14118
H 52	0.22984	0.00000	0.76671	0.00344	0.77016	-0.00424
C 53	-0.35481	1.99900	4.33285	0.02297	6.35481	0.13738
H 54	0.22973	0.00000	0.76691	0.00336	0.77027	-0.00400

```
   C 55   -0.14642      1.99877     4.12633    0.02131     6.14642     0.01339
   C 56   -0.37623      1.99898     4.35418    0.02306     6.37623     0.07878
   H 57    0.22936      0.00000     0.76735    0.00329     0.77064    -0.00245
   C 58   -0.41795      1.99899     4.39469    0.02426     6.41795    -0.01836
   H 59    0.22980      0.00000     0.76680    0.00340     0.77020     0.00042
   C 60   -0.13056      1.99877     4.11040    0.02139     6.13056     0.08899
   C 61   -0.14481      1.99874     4.12492    0.02115     6.14481     0.05425
   C 62   -0.17339      1.99873     4.15212    0.02253     6.17339    -0.00327
   C 63   -0.13500      1.99874     4.11527    0.02099     6.13500     0.05002
   C 64   -0.16070      1.99873     4.14019    0.02177     6.16070     0.01660
   C 65   -0.15783      1.99873     4.13774    0.02135     6.15783     0.02275
  Cs 66    0.92755     53.98666     0.03088    0.05492    54.07245     0.01093
 ===============================================================================
 * Total *  0.00000    403.88450   184.88203    1.23347   590.00000     2.00000
```

Table S18. Absolute energies of all systems PBE0/def2-TZVP(*metal*)//cc-pVDZ(C,H,O) level of theory.

Compound	Energy, a.u.
**1**-K-*full*	-5823.0050863012
**1H**-K-*full*	-5822.9412782329
**1H**-K-*small*	-3974.1414602132
**1**-Li-*small*	-1605.1443852236
**1**-Na-*small*	-2223.8839720665
**1**-K-*small*	-3974.1571072552
**1**-Rb-*small*	-1671.5057489719
**1**-Cs-*small*	-1655.7565934532

EDA analysis of 1-M-small systems

The bonding between bowl-shaped fragments and positively charged alkali metal belts (see Fig. S11 for details) was further investigated by the energy decomposition analysis (EDA) developed by Morokuma and by Ziegler and Rauk.[18] For this purpose, single-point calculations were performed by the ADF program package[19] with the same functional. All atoms were described by uncontracted Slater-type orbitals (STOs) with TZ2P quality as basis functions.[20] An auxiliary set of s, p, d, and f STOs was used to fit the molecular densities and to represent the Coulomb and exchange potentials accurately in each SCF cycle.[21] Scalar relativistic effects have been taken into account by ZORA. Further details on the EDA can be found in literature.[22]

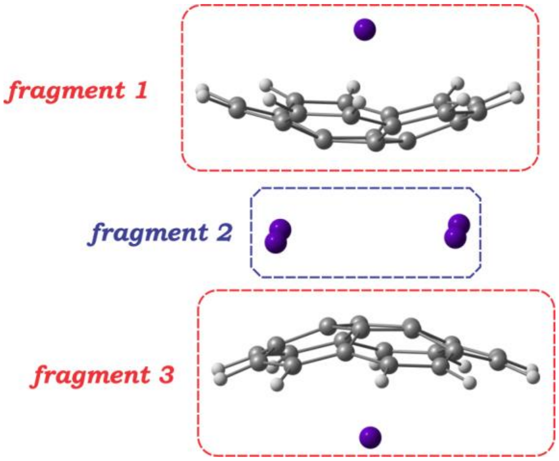

Figure S11. EDA fragmentation scheme in **1**-M-*small* models, where M = Li–Cs.

Influence of bowls sliding on magnetic properties

In order to provide additional arguments on stabilization of the ferromagnetic component of magnetic coupling between bowl-shaped radicals in target supramolecular aggregates due to bowl sliding with respect to each other, the modified **1**-Li-*small* system was considered (hereafter called **1**-Li-*small*-iso). In this new model, two bowl-fragments were placed right on top of each other (in other words, in a non-sliding fashion), whereas the distance d_1 was kept exactly the same as in the fully relaxed **1**-Li-*small* system. Thus, the influence of the bowl shift on magnetic coupling between two bowls was excluded. Magnetic coupling in this system was calculated at the same level of theory, using broken-symmetry DFT approach (BS-PBE0/TZVP/ZORA).

Table S19. Results of modelling of magnetic coupling (2*J*, in cm^{-1}) in **1**-M-*small* and **1H**-M-*small* systems (M = K, Cs) at different levels of theory.

System	Method

	BS-PBE0	MRMP2	Exp.
**1H**-K-*small*	+1.50	-6.44	-8.0
**1**-K-*small*	-2.62	-54.43	
**1H**-Cs-*small*	+3.84	+0.002	
**1**-Cs-*small*	-2.39	-0.01	

V. References

1 N. V. Kozhemyakina, J. Nuss, M. Jansen, *Z. Anorg. Allg. Chem.*, 2009, **635**, 1355–1361.

2 (a) G. Mehta, G. Panda, *Tetrahedron Lett.*, 1997, **38**, 2145–2148; (b) L. T. Scott, P.-C. Cheng, M. M. Hashemi, M. S. Bratcher, D. T. Meyer, H. B. Warren, *J. Am. Chem. Soc.*, 1997, **119**, 10963–10968; (c) A. Sygula, G. Xu, Z. Marcinow, P. W. Rabideau, *Tetrahedron*, 2001, **57**, 3637–3644.

3 B. Bleaney, K. D. Bowers, *Proc. R. Soc. London, Ser. A*, 1952, **214**, 451–465.

4 SADABS **2001**, Bruker AXS.

5 O. V. Dolomanov, L. J. Bourhis, R. J. Gildea, J. A. K. Howard, H. Puschmann. *J. Appl. Crystallogr.*, 2009, **42**, 339–341.

6 (a) G. M. Sheldrick, *Acta Crystallogr.*, 2008, **A64**, 112–122; (b) G. M. Sheldrick, *Acta Crystallogr.*, 2015, **C71**, 3–8.

7 (a) P. van der Sluis, A. L. Spek, *Acta Crystallogr*., 1990, **A46**, 194–201; (b) A. L. Spek, *Acta Crystallogr.*, 2015, **C71**, 9–18.

8 A. L. Spek, *Acta Crystallogr*., 2009, **D65**, 148–155.

9 A. A. Granovsky, Firefly, v.8.0.0, http://classic.chem.msu.su/gran/gamess/index.html

10 F. Neese, *ORCA*; University of Bonn: Bonn, Germany, **2009**.

11 (a) D. A. Pantazis, X.-Y. Chen, C. R. Landis and F. Neese, *J. Chem. Theory Comput.*, 2008, **4**, 908–919; (b) M. Buhl, C. Reimann, D. A. Pantazis, T. Bredow and F. Neese, *J. Chem. Theory Comput.*, 2008, **4**, 1449–1459.

12 F. Neese, F. Wennmohs, A. Hansen and U. Becker, *Chem. Phys.*, 2009, **356**, 98–109.

13 (a) F. Weinhold and C. A. Landis, *Valency and Bonding: A Natural Bond Orbital Donor−Acceptor Perspective*, Cambridge University Press: Cambridge, 2005; (b) A. E. Reed, L. A. Curtiss and F. Weinhold, *Chem. Rev*., 1988, **88**, 899–926.

14 E. D. Glendening, J. K. Badenhoop, A. E. Reed, J. E. Carpenter, J. A. Bohmann, C. M. Morales and F. Weinhold, *NBO 6.0*; University of Wisconsin: Madison, Wisconsin, **2013**.

15 K. Yamaguchi, Y. Takahara, T. Fueno *in*: V.H. Smith (Ed.) *Applied Quantum Chemistry*, Reidel, Dordrecht, **1986**, pp 155.

16 A. A. Granovsky, *J. Chem. Phys*., 2011, **134**, 214113.

17 H. Nakano, *J. Chem. Phys*., 1993, **99**, 7983–7992.

18 (a) K. J. Morokuma, *Chem. Phys*., 1971, **55**, 1236–1244; (b) T. Ziegler, A. Rauk, *Inorg. Chem*., 1979, **18**, 1558–1565; (c) T. Ziegler, A. Rauk, *Inorg. Chem*., 1979, **18**, 1755–1759.

19 (a) C. Fonseca Guerra, J. G. Snijders, G. te Velde, E. J. Baerends, *Theor. Chem. Acc.*, 1998, **99**, 391–403; (b) *ADF2008.01*, SCM, Vrije Universiteit, Amsterdam, Netherlands, http://www.scm.com; (c) G. te Velde, F. M. Bickelhaupt, E. J. Baerends, C. Fonseca Guerra, S. J. A. van Gisbergen, J. G. Snijders, T. Ziegler, *J. Comput. Chem.*, 2001, **22**, 931–967.

20 J. G. Snijders, E. J. Baerends, P. Vernooijs, *At. Data Nucl. Data Tables*, 1982, **26**, 483–574.

21 J. Krijn, E. J. Baerends, *Fit Functions in the HFS-Method*, **1984**, Internal Report (In Dutch), Vrije Universiteit, Amsterdam, Netherlands.

22 (a) M. Lein, G. Frenking, in *Theory and Application of Computational Chemistry*, ed. Dekstra, C. **2005**; (b) M. von Hopffgarten, G. Frenking, in *Computational Inorganic and Bioinorganic Chemistry*, ed. E. Solomon, **2009**, pp. 3–15; (c) M. Lein, A. Szabo, A. Kovac, G. Frenking, *Faraday Discuss*., 2003, **124**, 365–378; (d) G. Frenking, K. Wichmann, N. Frohlich, C. Loschen, M. Lein, J. Frunzke, V. M. Rayon, *Coord. Chem. Rev.*, 2003, **238-239**, 55–82; (e) A. Krapp, F. M. Bickelhaupt, G. Frenking, *Chem. Eur. J.*, 2006, **12**, 9196–9216; (f) A. Krapp, G. Frenking, G. *J. Comput. Chem*., 2007, **28**, 15–24; (g) M. von Hopffgarten, G. Frenking, *WIREs Comput. Mol. Sci*., 2012, **2**, 9196–9216.
